# Semiparametric Panel Count Model, With Applications to Signal Detection in Post‐Market Drug Surveillance Systems

**DOI:** 10.1002/sim.70649

**Published:** 2026-07-09

**Authors:** Yizhao Zhou, Ao Yuan, Ming Tan

**Affiliations:** ^1^ Department of Biostatistics, Bioinformatics & Biomathematics Georgetown University Washington DC USA

**Keywords:** covariate information, drug safety, semiparametric panel count model, signal detection

## Abstract

Panel count data occurs in a wide variety of applications, ranging from biomedical research to business, such as the number of accidents, product defects, and insurance claims. For such data under the FDA investigation, millions of reported adverse events (AEs) associated with thousands of drugs are monitored in the post‐market drug safety surveillance systems worldwide. Evaluating the AEs of the associated drugs is an important public health concern and motivates our method. One statistical challenge in such systems is handling the excessive number of zero AE counts. Most existing methods utilize Poisson count models that cannot incorporate covariates nor account for the excessive zero counts adequately. This article proposes a novel semiparametric nonhomogeneous panel count model to detect AE signals by accounting for covariates, background AE occurrences, and excessive zero counts. The model is estimated using the Expectation‐Maximization (EM) algorithm iteratively, where in each M‐step, the maximization of the nonparametric component is reformulated as an optimization problem, as in the isotonic regression. The strong consistency and the asymptotic distributions of the estimators are formally derived. We conduct extensive simulation studies to evaluate the finite sample performance of the proposed method and to demonstrate the apparent advantage of the proposed method in signal detection with high power, high specificity, and sensitivity. We apply the method to a VigiBase dataset to detect the AE signals as an application of the proposed method.

## 1 Introduction

Panel count data occur in a wide range of applications, from scientific investigation and evidence‐based policy research to business, including the number of accidents, product defects, and insurance claims. One application of great public health importance is in the (e.g., the FDA's) post‐market drug safety surveillance systems, where millions of reported adverse events (AEs) associated with thousands of drugs are monitored. As pre‐marketing studies generally have minimal power to detect all the potential drug AEs [1, 2] due to their limited sample sizes, special patient selection criteria, and care settings. Therefore, it is essential for regulatory agencies and pharmaceutical companies to monitor post‐market drug safety and identify signals in the surveillance systems. However, inadequate methods used may have severe consequences, including withdrawal of the product from the market. There are currently several post‐market surveillance systems worldwide, including the VigiBase and the FDA Adverse Event Reporting System (FAERS). The individual safety reports in FAERS and VigiBase contain covariates such as patient demographics, drug/substance information, the seriousness of the case, and drug intake duration. It is of great importance to identify AEs with such covariate information incorporated to get more accurate reporting rates for a specific drug or identify the drugs with high reporting rates for specific AEs in the post‐surveillance systems.

Such systems contain a large number of subjects taking different drugs over many years. If a drug on the market has shown unacceptable toxicities, the drug may have to be taken off the market for obvious public health reasons. However, it also potentially limits treatment options for some patients. A unique feature of such data is that the background noise is strong, with limited information collected. Moreover, after collapsing data into the form of an I×J table, a large number of cell counts are zeros since a common AE for one drug might be rare for another drug. For example, the FAERS reports in the 4th quarter of 2019 include about 1657 drugs and 9262 types of AEs where 98.79% of cell counts in drugs by AE types are zeros, although there is a large collective count of AEs. Another feature is that such systems contain patient demographics for which could be adjusted to remove imbalances resulting from heterogeneous patients' characteristics from one drug to another. This motivates us to develop a novel model capable of accurately detecting true signals in such large and complex systems while incorporating essential patient characteristics, including demographic information.

Detecting AE signals in such systems has been a long‐standing statistical problem. The WHO drug monitoring center has used the K index, which is the root of Pearson's $\chi^{2}$ test statistic with Yates's correction [3, 4]. The approach is based on the principle that the most significant drug‐AE combinations have the greatest K and are easily understood. It is not hard to compute all the AEs in the 1960s and 1970s when the database is not large. However, they do not have definite thresholds of the K index to define potential signals, which is only determined by previous experience and comparing combinations when applying this index. The Proportional Reporting Ratio (PRR) approach developed by Evans et al. [5] reduces the vagueness in thresholds by quantifying the judgment of signals based on the PRR, the value of chi‐squared, and the absolute number of reports. PRR is a straightforward approach based on the proportionate approach in a 2×2 table. PRR is simple to calculate and interpret. However, if we view the 2×2 table as one in a case‐control study, which means the drug of interest as a case group and other drugs as a control group, Reporting Odds Ratio (ROR) would have been more suitable than PRR as the ORs' ability is close to relative risk in case‐control studies when the event is rare [6, 7]. The ROR measure does remove part of the bias of PRR. However, it suffers from critical deficiencies with PRR, such as inflated overall type I error generated from multiple comparisons because of large drug‐event combinations.

Indeed, the likelihood ratio test (LRT) based approach proposed by Huang et al. [8] controls the overall type I error in the signal screening process by a step‐down process. LRT assumes the cell counts of the drug‐AE combination tables follow Poisson distributions. However, excessive zeros in the database violate this assumption. Huang et al. [9] improved the LRT‐based approach by assuming the cell counts follow a zero‐inflated Poisson (ZIP) distribution to handle the zero counts issue. Both LRT and ZIP LRT methods only consider the drug combinations for a single particular drug at one time. To monitor a class of drugs, Huang et al. [10] extended the LRT approach for signals, including collections of drugs (or AEs), by introducing a weight matrix to indicate if drugs belong to the same drug class. Simulation studies demonstrate that LRT‐based approaches control both type I error and false discovery rate (FDR). In addition, these LRT‐based approaches allow stratified analysis if the number of strata is small. However, they also have several limitations: (1) their p values are determined by an empirical null distribution obtained by Monte Carlo simulation. It would not be practical and time‐consuming when drugs included are large when Monte Carlo simulation needs to repeat thousands of times in one process; (2) LRT‐based approach is still built on the PRR measure, so it shares the limitation of PRR mentioned above; (3) the handling of excessive zero counts by the ZIP model is not satisfactory: ZIP LRT dealt with the excessive zero counts, but the assumption is questionable since excessive large collective counts of some drugs are still not accounted for by a Poisson distribution, which is a severe problem; (4) the model and analysis is for each AE/drug separately without considering the collective roles of the AEs on the drugs; and (5) stratified analysis is not able to incorporate covariates information, especially for continuous variables or when the number of covariates is large.

Furthermore, Bayesian data mining has also been used [11]. Bate et al. [12] applied the Bayesian Confidence Propagation Neural Network (BCPNN) [13] to the WHO database with a one‐layer model based on Information Component(IC) measure. The Multi‐Item Gamma Poisson Shrinker (MGPS) [11] approach assumes the prior on relative report rate (RRR) is a mixture of two gamma distributions and the number of drug‐AE combination follows the Poisson distribution, and then compares the Empirical Bayesian Geometric Mean (EGBM) scores with thresholds. Stratified analyses are used to control potential confounding factors. Norén et al. [14] extends the BCPNN approach to allow for more complex quantitative associations by assuming cell counts of the 2×2 table following multinomial instead of binomial distribution in the original paper of Bate et al. [12]. They also propose a Mantel‐Haenszel‐type adjustment for the IC to control potential confounders. The biggest issue with the Bayesian approach is that it depends on prior distributions and does not address the zero‐inflated problems.

Existing methods have assumed either a homogeneous Poisson model and its variant, the Zip‐Poisson model, as the Poisson model is not accurate due to the large number of zero counts in $y_{ij}$s. Indeed, the Zip‐Poisson parametric model has improved the Poisson model, but it continues to have several serious problems:

a. The Zip‐Poisson model assumes the counts are from a mixture of zero and Poisson distribution, but the excessively large $y_{.j}-y_{ij}$ may not follow the Poisson either; in fact, the parametric model lacks the needed robustness against the noisy safety signals;
b. The counts for each drug are modeled separately, while joint modeling of a class of drugs would be more meaningful by borrowing information from each drug;
c. Each AE is modeled separately, while joint modeling of AEs would be more meaningful by borrowing information from each AE; and
d. Most importantly, the existence that may impact the signal

To overcome the aforementioned issues, we propose a semiparametric panel count model to detect safety signals that capture higher reported occurrences than background occurrences in surveillance systems robustly. The method treats each zero as a result of a latent indicator being from Poisson or not, and both categorical and continuous covariates, such as age, gender, and weights, are incorporated into the model. Since the patient population in such systems is known to be very heterogeneous, such modeling has the potential to bring significantly improved accuracy in signal detection by accounting for such heterogeneity. We show that the new method is able to expand the capabilities of current methods and reduce the bias from RR‐based approaches. In addition, this method utilizes all the data jointly, that is, system‐wide, instead of separately for each drug, accounts for both the extra‐large marginal total counts for each drug, and the nonzero counts to the extent that the ZIP model is not able to. We use a nonparametric component to adjust for the noisy background of the surveillance data. We formulate signal detection as hypothesis testing based on the estimated model, adopting the Holm‐Bonferroni approach to control the family‐wise error rate (FWER) in the signal detection process.

The remainder of this article is organized as follows. The proposed semiparametric model is formulated in Section 2, and the model parameters are estimated with an iterative algorithm in Section 3. We derive asymptotic properties of the estimators in Section 4. In Section 5, we discuss hypothesis tests of signal detection. Simulations (Section 6) are carried out to evaluate the proposed method's finite sample properties and compare them with existing methods. The method is then applied to a VigiBase dataset in Section 7. We conclude with a summary and remarks (Section 8). The complete simulation results and technical details, such as proofs of asymptotic theories, are given in the Supporting Information.

## 2 Statistical Model Formulation

To investigate the J drugs or biologics (e.g., adriamycin, remdesivir, COVID‐19 vaccines) and their corresponding I AEs (e.g., hives, headache, fatigue, etc.), for example, from a surveillance system, let $y_{ij}$ be the reported occurrences of the i‐th AE for the jth drug. Then $\left\{y_{ij}\right\}$ constitute a I×J table of counts. For each fixed drug j, $y_{ij}$'s can be viewed as the number of type i events in the “time” interval $\left(0,t_{i}\right]$. Throughout this article, we assume the observed data is of the form $\left\{(t_{i},y_{ij},x_{ij}):i=1,\ldots ,I;j=1\ldots ,J,\right\}$, where $t_{i}$ is a “time” variable, $x_{ij}\in R^{d}$ is the corresponding covariate vector. Without loss of generality, we assume $t_{i}\in [0,1]$. Denote $y_{i}$ for the length $k_{i}$ vector of all the nonzero counts of $\left\{y_{ij}:j=1,\ldots ,J\right\}$, $x_{i}=(x_{i1},\ldots ,x_{iJ})$ for the corresponding covariates (e.g., age, gender) where the orders of the components in $y_{i}$ match those in $x_{i}$. It is well recognized that $y_{ij}$'s contain too many zero counts to follow a Poisson distribution. So often the nonzero counts $y_{i}$'s are assumed to be from a Poisson distribution. For the zero counts, let $\delta_{ij}$ be the latent variable indicating if the zero is from the Poisson distribution or not, and $\delta_{ij}=1$ if the zero count is from the same Poisson distribution as the $y_{i}$'s, otherwise $\delta_{ij}=0$. For fixed i, denote $\delta_{i}$ be the vector of all the $\delta_{ij}$'s thus $dim(\delta_{i})=J-k_{i}$. Existing approaches have assumed a homogeneous Poisson model for the AE rates, which is questionable since the zero counts may be too excessive, and $y_{i.}-y_{ij}$ can often be large count values up to 10,000 that the ZIP model no longer follows.

Consequently, assuming all the AEs are independent, we formulate the following nonhomogeneous Poisson process $y_{ij}=y_{ij}(t_{i})$ for counts in “time” interval $\left(0,t_{i}\right]$ with conditional mean

$$E(y_{ij}(t_{i})|x_{ij})=G(t_{i})\exp(\beta^{⊺}x_{ij}),\;\;\;\;\;\;\;\;\;G\in 𝒢,$$

where 𝒢={monotone increasing functions on [0,1] with 0≤G(·)≤1.}. The constraint $0\leq G(t_{i})\leq 1$ is for the identifiability of the model. The nonparametric component G(·) is to capture the background occurrence of AEs in the panel, which varies with different AEs.

## 3 The Likelihood Function

To estimate the model parameters, we first include the latent data $\delta_{i}$'s, construct a joint model under the “complete data” $D_{I,J}=\{(t_{i},y_{ij},x_{i},\delta_{i}):i=1,\ldots ,I;j=1,\ldots ,J\}$. Without loss of generality, we assume that the first $k_{i}$ cells are non‐zeros for a fixed i. Then conditioning on $\left(t_{i},x_{ij},\delta_{i}\right)$, omitting irrelevant terms, the mass function of $y_{i}$ is

(1) $$\begin{array}{ll} & p(y_{i}|t_{i},x_{i},\delta_{i};\beta ,G)=\left(\overset{k_{i}}{\underset{j=1}{\prod }}\frac{{\left(G(t_{i})\exp(\beta^{⊺}x_{ij})\right)}^{y_{ij}}}{y_{ij}!}\right)\\ & \;\times \exp\left\{-G(t_{i})\left[\overset{k_{i}}{\underset{j=1}{\sum }}\exp(\beta^{⊺}x_{ij})+\overset{J}{\underset{j=k_{i}+1}{\sum }}\delta_{ij}\exp(\beta^{⊺}x_{ij})\right]\right\}. \end{array}$$

The mass function of the observed data is more involved. Let $y={\left(y_{1},\ldots ,y_{J}\right)}^{⊺}$ and (t,y,x) be an i.i.d. copy of $\left(t_{i},y_{i},x_{i}\right)$. Let $\lambda =P(\delta_{j}=1)$, denote $\exp(\beta^{⊺}x)={\left(\exp(\beta^{⊺}x_{k+1},\ldots ,\exp(\beta^{⊺}x_{J})\right)}^{⊺}$, $r={\left(r_{k+1},\ldots ,r_{J}\right)}^{⊺}$ with $r_{j}=0$ or 1, $|r|=\sum_{j=k+1}^{J}r_{j}$, and θ=(β,λ). The mass function of y conditioning on (t,x) is, with λ as a nuisance parameter,

(2) $$\begin{array}{ll} & p(y|t,x;\theta ,G)=\left(\overset{k}{\underset{j=1}{\prod }}\frac{{\left(G(t)\exp(\beta^{⊺}x_{j})\right)}^{y_{j}}}{y_{j}!}\right)\\ & \;\exp\left\{-G(t)\overset{k}{\underset{j=1}{\sum }}\exp(\beta^{⊺}x_{j})\right\}\\ & \;\times \overset{J-k}{\underset{|\text{r}|=0}{\sum }}\exp\left\{-G(t)r^{⊺}\exp(\beta^{⊺}x)\right\}\lambda^{\left|\text{r}\right|}{\left(1-\lambda \right)}^{J-k-|\text{r}|}. \end{array}$$

The likelihood (2) for the observed data is not easy to work with due to the summation structure on the right term, so we use the likelihood (1) based on the “complete data” data $D_{I,J}$ as

$$\begin{array}{ll} & L_{I,J}(\beta ,G)=\overset{I}{\underset{i=1}{\prod }}\left(\overset{k_{i}}{\underset{j=1}{\prod }}\frac{{\left(G(t_{i})\exp(\beta^{⊺}x_{ij})\right)}^{y_{ij}}}{y_{ij}!}\right)\\ & \;\times \exp\left\{-G(t_{i})[\overset{k_{i}}{\underset{j=1}{\sum }}\exp(\beta^{⊺}x_{ij})+\overset{J}{\underset{j=k_{i}+1}{\sum }}\delta_{ij}\exp(\beta^{⊺}x_{ij})]\right\}. \end{array}$$

Recall $y_{i.}=\sum_{j=1}^{J}y_{ij}$. The corresponding log‐likelihood is, omitting some constant independent parameter of interest,

(3) $$\begin{array}{ll} \ell_{I,J}(\beta ,G) & =\overset{I}{\underset{i=1}{\sum }}\left(\overset{k_{i}}{\underset{j=1}{\sum }}y_{ij}\left[\log G(t_{i})+\beta^{⊺}x_{ij}\right]\right.\\ & \;\left.-G(t_{i})[\overset{k_{i}}{\underset{j=1}{\sum }}\exp(\beta^{⊺}x_{ij})+\overset{J}{\underset{j=k_{i}+1}{\sum }}\delta_{ij}\exp(\beta^{⊺}x_{ij})]\right). \end{array}$$

Denote B for the space for β, we estimate the true parameter $\left(\beta_{0},G_{0}\right)$ by the semiparametric maximum likelihood estimator $\left(\hat{\beta },Ĝ\right)$,

(4) $$(\hat{\beta },Ĝ)=\underset{\;(\beta ,G)\in (B,𝒢)}{\arg\;\max}\ell_{I,J}(\beta ,G).$$

However, $\left(\hat{\beta },Ĝ\right)$ cannot be directly computed, as $\left\{\delta_{i}:i=1,\ldots ,I\right\}$ are missing, so we use an iterative version of the EM‐algorithm [15] to handle it as below.

*Estimation algorithm*. Given starting value $\left(\beta^{\left(0\right)},G^{\left(0\right)}\right)$, compute the next step estimate $\left(\beta^{\left(1\right)},G^{\left(1\right)}(\cdot )\right)$. Generally, given $\left(\beta^{\left(r\right)},G^{\left(r\right)}\right)$, $\left(\beta^{\left(r+1\right)},G^{\left(r+1\right)}\right)$ is updated in the M‐step and E‐step as follows.

*E‐step*. Let $D_{I,J}^{0}$ be $D_{I,J}$ without the $\delta_{j}$'s, which is the set of observed data. Given $\left(\beta^{\left(r\right)},G^{\left(r\right)}\right)$, let

$$H_{I,J}(\beta ,G|\beta^{\left(r\right)},G^{\left(r\right)})=E_{\left(\beta^{\left(r\right)},\lambda ,G^{\left(r\right)}\right)}\left(\ell_{I,J}(\beta ,G)|D_{I,J}^{0},\beta^{\left(r\right)},G^{\left(r\right)}\right),$$

where the expectation is with respect to the missing $\delta_{j}$'s conditioning on the observed data, as if $\left(\beta^{\left(r\right)},G^{\left(r\right)}\right)$ were the true parameters. By (3),

$$\begin{array}{ll} & H_{I,J}(\beta ,G|\beta^{\left(r\right)},G^{\left(r\right)})=\overset{I}{\underset{i=1}{\sum }}\left(\overset{k_{i}}{\underset{j=1}{\sum }}y_{ij}\left[\log G(t_{i})+\beta^{⊺}x_{ij}\right]\right.\\ & \;-G(t_{i})\left[\overset{k_{i}}{\underset{j=1}{\sum }}\exp(\beta^{⊺}x_{ij})\right.\left.\left.+\overset{J}{\underset{j=k_{i}+1}{\sum }}E(\delta_{ij}|D_{I,J}^{0},\beta^{\left(r\right)},G^{\left(r\right)})\exp(\beta^{⊺}x_{ij})\right]\right). \end{array}$$

To compute $E(\delta_{ij}|D_{I,J}^{0},\beta^{\left(r\right)},G^{\left(r\right)}):=\delta_{ij}^{\left(r\right)}$, let $m_{i}$ be the expected number of zero counts for the observed $k_{i}$ nonzero counts in the same Poisson(μ) distribution, then $k_{i}+m_{i}$ is the total number of observations in the Poisson experiment, so the number of expected zero counts $m_{i}\approx (k_{i}+m_{i})\exp(-\mu )$, or $m_{i}\approx k_{i}\exp(-\mu )/\left(1-\exp(-\mu )\right)$, and $E(\delta_{ij}|\mu )=m_{i}/(J-k_{i})\approx k_{i}\exp(-\mu )/[(I-k_{i})\left(1-\exp(-\mu )\right)]$. However, in our case, exp(−μ) changes with individuals due to the varying covariates, so we use the following value to approximate it.

$$\begin{array}{ll} \exp(-\mu_{i}^{\left(r\right)}) & :=\frac{k_{i}}{k_{i}+m_{i}}\frac{1}{k_{i}}\overset{k_{i}}{\underset{j=1}{\sum }}\exp\left\{-G^{\left(r\right)}(t_{i})\exp(\beta^{(r)⊺}x_{ij})\right\}\\ & \;+\frac{m_{i}}{k_{i}+m_{i}}\frac{1}{J-k_{i}}\overset{J-k_{i}}{\underset{j=1}{\sum }}\exp\left\{-G^{\left(r\right)}(t_{i})\exp(\beta^{(r)⊺}x_{ij})\right\}\\ & :=\frac{k_{i}}{k_{j}+m_{i}}A_{i}^{\left(r\right)}+\frac{m_{i}}{k_{i}+m_{i}}B_{i}^{\left(r\right)}=\left(1-\exp(-\mu_{i}^{\left(r\right)})\right)\\ & \;A_{i}^{\left(r\right)}+\exp(-\mu_{i}^{\left(r\right)})B_{i}^{\left(r\right)}. \end{array}$$

The above gives $\exp(-\mu_{i}^{\left(r\right)})=A_{i}^{\left(r\right)}/(1+A_{i}^{\left(r\right)}-B_{i}^{\left(r\right)})$, and

$$\begin{array}{cc} \delta_{ij}^{\left(r\right)} & =E(\delta_{sj}|D_{I,J}^{0},\beta^{\left(r\right)},G^{\left(r\right)})\\ & =k_{i}\exp(-\mu_{i}^{\left(r\right)})/[(J-k_{i})\left(1-\exp(-\mu_{i}^{\left(r\right)})\right)]\\ & =\frac{k_{i}}{J-k_{i}}\frac{A_{i}^{\left(r\right)}}{1-B_{i}^{\left(r\right)}}. \end{array}$$

Plugging the $\delta_{ij}^{\left(r\right)}$'s into the above‐expected log‐likelihood, we get

$$\begin{array}{ll} & H_{I,J}(\beta ,G|\beta^{\left(r\right)},G^{\left(r\right)})=\overset{I}{\underset{i=1}{\sum }}\left(\overset{k_{i}}{\underset{j=1}{\sum }}y_{ij}\left[\log G(t_{i})+\beta^{⊺}x_{ij}\right]\right.\\ & \;-G(t_{i})\left[\overset{k_{i}}{\underset{j=1}{\sum }}\exp(\beta^{⊺}x_{i})\right.\left.\left.+\overset{J}{\underset{j=k_{i}+1}{\sum }}\delta_{ij}^{\left(r\right)}\exp(\beta^{⊺}x_{ij})\right]\right). \end{array}$$

*M‐step*. Compute

$$(\beta^{\left(r+1\right)},G^{\left(r+1\right)})=\underset{\;(\beta ,G)\in (B,𝒢)}{\arg\;\max}H_{I,J}(\beta ,G|\beta^{\left(r\right)},G^{\left(r\right)}).$$

In the above, the maximization over G is nontrivial. Our idea is to convert the complex optimization problem to a sequence of isotonic regression procedures. Such an approach has proved successful in semiparametric models for subgroup analysis in Zhou et al. [16] and Yuan et al. [17]. Here, our model is more complicated, and we describe the algorithm below.

*Computation of* $G^{\left(r+1\right)}(\cdot )$. In particular, we can first fixed $\beta^{\left(r\right)}$, maximizing over G(·) to get $G^{\left(r+1\right)}(\cdot )$, which is of the form, for some $d_{i}^{\left(r\right)}$'s,

$$G^{\left(r+1\right)}=\underset{\;G\in 𝒢}{\arg\;\max}\;\overset{I}{\underset{i=1}{\sum }}\left(y_{i.}\log G(t_{i})-d_{i}^{\left(r\right)}G(t_{i})\right),$$

where

$$d_{i}^{\left(r\right)}=\overset{k_{i}}{\underset{j=1}{\sum }}\exp(\beta^{(r)⊺}x_{ij})+\overset{J}{\underset{j=k_{i}+1}{\sum }}\delta_{ij}^{\left(r\right)}\exp(\beta^{(r)⊺}x_{ij}).$$

For this, without loss of generality, we assume the $t_{i}$'s are arranged in increasing order, rewrite the above as, with $h_{i}^{\left(r\right)}=y_{i.}/d_{i}^{\left(r\right)}$,

$$\begin{array}{ll} G^{\left(r+1\right)} & =\underset{\;G\in 𝒢}{\arg\;\min}\;\overset{I}{\underset{i=1}{\sum }}\left(-h_{i}^{\left(r\right)}\log G(t_{i})+G(t_{i})\right)d_{i}^{\left(r\right)}.\\ & =\underset{\;G\in 𝒢}{\arg\;\min}\;\overset{I}{\underset{i=1}{\sum }}(h_{j}^{\left(r\right)}\log h_{i}^{\left(r\right)}-h_{i}^{\left(r\right)}\log G(t_{i})-h_{i}^{\left(r\right)}+G(t_{i}))d_{i}^{\left(r\right)}\\ & =\underset{\;G\in 𝒢}{\arg\;\min}\;\overset{I}{\underset{i=1}{\sum }}\Delta_{\Phi }(h_{i}^{\left(r\right)},G(t_{i}))d_{i}^{\left(r\right)}, \end{array}$$

where $\Delta_{\Phi }(u,v)=\Phi (u)-\Phi (v)-(u-v)\phi (v)=u\log u-v\log v-(u-v)(\log v+1)=u\log u-u\log v-u+v$, and $\Phi (u)=u\log u,u\in R^{+}$. The first derivative of Φ is given by ϕ(u)=logu+1. Φ(·) is convex on $R^{+}$, so by theorem 1.5.1 and example 1.5.1 in Robertson et al. [18], the above minimization is written as

(5) $$G^{\left(r+1\right)}=\underset{\;G\in 𝒢}{\arg\;\min}\;\overset{I}{\underset{i=1}{\sum }}d_{i}^{\left(r\right)}{\left(h_{i}^{\left(r\right)}-G(t_{i})\right)}^{2}.$$

Then the above algorithm is converted into an isotonic regression problem, and can be computed using the R‐package isotonic [19, 20]. Then, fix $G^{\left(r+1\right)}(\cdot )$, maximizing over β to get $\beta^{\left(r+1\right)}$, the iteration goes on until convergence of the sequence, and the final values are treated as the MLE $\left(\hat{\beta },Ĝ\right)$.

The convergence of the above iterative procedure is implied by noting

$$\begin{array}{cc} H_{I,J}(\beta^{\left(r\right)},G^{\left(r\right)}|\beta^{\left(r\right)},G^{\left(r\right)}) & \leq H_{I,J}(\beta^{\left(r\right)},G^{\left(r+1\right)}|\beta^{\left(r\right)},G^{\left(r\right)})\\ & \leq H_{I,J}(\beta^{\left(r+1\right)},G^{\left(r\right)}|\beta^{\left(r\right)},G^{\left(r\right)}) \end{array}$$

and the ascending property of the EM algorithm. So the sequence $\left\{(\beta^{\left(r\right)},G^{\left(r\right)}\right\}$ will converge to at least some local maxima, and multiple starting points may be required to locate the global maxima, just like the EM algorithm without iteration. In our implementation, we initialize the parametric component, denoted by $\beta^{\left(0\right)}$, using a simple parametric working model that ignores the nonparametric component by setting G(·)≡1. Given this initial choice of $\beta^{\left(0\right)}$, we obtain an initial estimate $G^{\left(0\right)}$ with EM algorithm described above.

## 4 Theoretical Properties

Theorem 1 below demonstrates strong consistency of the estimators $\left(\hat{\beta },Ĝ\right)$; Theorem 2 below gives asymptotic normality and efficiency of the Euclidean component $\hat{\beta }$; Theorem 3 gives the asymptotic distribution of Ĝ(·) in a closed‐form; and Theorem 4 displays the general asymptotic distribution of the derivatives of Ĝ(·).

Let $\left(\theta_{0},G_{0}\right)$ be the ‘true’ parameters generating the observed data. We list the following regularity conditions:

C1. $\beta_{0}\in B$, a compact set.
C2. The support of X is compact.
C3. $G_{0}\in 𝒢$ and $G_{0}(\cdot )$ is continuous.
C4. $\sup_{G\in 𝒢}\sup_{t\in [0,1]}G(t)<\infty$.
C5. On a small neighborhood ${𝒢}_{\eta }=\{G:\sup_{t\in [0,1]}|G(t)-G_{0}(t)|\leq \eta \}$ of $G_{0}$ for some small η>0, $\sup_{G\in {𝒢}_{\eta }}E[1/G^{2}(T)]<\infty$.
C6. $0<\lambda_{0}<1$.

Theorem 1 *Assume C*1*–C*6, *then for fixed* J, *as* I→∞,

$$\hat{\beta }\overset{a.s.}{\to }\beta_{0},\;\;\;\text{and}\;\;\;\;\underset{t\in [0,1]}{\sup}|Ĝ(t)-G_{0}(t)|\overset{a.s.}{\to }0.$$

*Denote* $\overset{D}{\to }$ *for convergence in distribution. Let* s:=(t,y,x), ℓ(θ,G|s)=logp(y|t,x;θ,G), *with* p(y|t,x;θ,G) *given in* (*2*), ${\dot{\ell }}_{\theta }(\theta ,G|s)=\partial \ell (\beta ,G|s)/\partial \theta$. *Theorem* *2* *below gives asymptotic normality and efficiency of the Euclidean component* $\hat{\beta }$. *The efficient score for estimating* $\theta_{0}$ *is given by* (*see Lemma* 2 *in the* Supporting Information)

$$\begin{array}{ll} \ell_{\theta }^{*}(\theta_{0},G_{0}|s) & ={\dot{\ell }}_{\theta }(\theta_{0},G_{0}|s)-A(s|\theta_{0},G_{0})h^{*}(t), \end{array}$$

$$\begin{array}{ll} & \;\text{where}\;A(s|\theta_{0},G_{0})=\overset{k}{\underset{j=1}{\sum }}\left[\frac{y_{j}}{G_{0}(t)}-\exp(\beta_{0}^{⊺}x_{j})\right] \end{array}$$

$$\begin{array}{ll} & \;-\frac{\sum_{|\text{r}|=0}^{J-k}\lambda_{0}^{\left|\text{r}\right|}{\left(1-\lambda_{0}\right)}^{J-k-|\text{r}|}\exp\left\{-G_{0}(t)r^{⊺}\exp(\beta_{0}^{⊺}x)\right\}r^{⊺}\exp(\beta_{0}^{⊺}x)}{\sum_{|\text{r}|=0}^{J-k}\lambda_{0}^{\left|\text{r}\right|}{\left(1-\lambda_{0}\right)}^{J-k-|\text{r}|}\exp\left\{-G_{0}(t)r^{⊺}\exp(\beta_{0}^{⊺}x)\right\}}, \end{array}$$

$$\begin{array}{ll} & \;\text{and}\;h^{*}(t)=\frac{E\left\{{\dot{\ell }}_{\theta }(\theta_{0},G_{0}|s)A(s|\theta_{0},G_{0})|t\right\}}{E\left\{A^{2}(s|\theta_{0},G_{0})|t\right\}}. \end{array}$$

*C*7. $I_{\beta }^{*}(\theta_{0},G_{0})$ *and* $I^{*}(\theta_{0},G_{0})$ *given in Theorem* *2* *are invertible*.

Theorem 2 *Assume C*1*–C*7, *then for fixed* J, *as* I→∞,

$$\sqrt{I}\left(\hat{\beta }-\beta_{0}\right)\overset{D}{\to }N(\text{0},{\left(I_{\beta }^{*}\right)}^{-1}),$$

*where* $I_{\beta }^{*}(\theta_{0},G_{0})$ *is the* β *‐block of* $I^{*}(\theta_{0},G_{0})=E_{\left(\theta_{0},G_{0}\right)}[\ell_{\theta }^{*}\ell_{\theta }^{*⊺}]$.

*Let* 𝔹(·) *be the two‐sided Brownian motion originating from zero: a mean zero Gaussian process on* R *with* 𝔹(0)=0, *and* $E{\left(𝔹(s)-𝔹(h)\right)}^{2}=|s-h|$ *for all* s,h∈R. *Theorem* *3* *gives the asymptotic distribution of* Ĝ(t) *under non‐vanishing condition of its derivative for each point* t.

*C*8. *Let* f(·) *be the density function of* t, ${Ġ}_{0}(t)=dG_{0}(t)/dt$. *Assume* f(t)>0 *and*
${Ġ}_{0}(t)>0$ *at the point* t *in Theorem* *3* *below*.
*C*9. $D_{1}(\cdot )$, $D_{2}(\cdot )$ *and* $\eta^{2}(\cdot )$ *given in Theorem* *3* *below are continuous at* t.

Theorem 3 *Assume C*1*–C*9, *then for fixed* J, *as* I→∞,

$$I^{1/3}\left(Ĝ(t)-G_{0}(t)\right)\overset{D}{\to }{\left(\frac{4\eta^{2}(t)D_{2}(t){Ġ}_{0}(t)}{D_{1}^{2}(t)f(t)}\right)}^{1/3}\underset{h}{\text{arg min}}\{𝔹(h)+h^{2}\}$$

*where* $\eta^{2}(t)=E[\epsilon^{2}|t]$, $\epsilon =y_{\cdot }/d-G_{0}(t)$, $y_{\cdot }=\sum_{j=1}^{J}y_{j}$, $d=\sum_{j=1}^{k}\exp(\beta_{0}^{⊺}x_{j})+\sum_{j=k+1}^{J}{\hat{\delta }}_{j}\exp(\beta_{0}^{⊺}x_{j})$, $D_{1}(t)=E[d|t]$ *and* $D_{2}(t)=E[d^{2}|t]$.

*In Theorem* *3*, *it is assumed that* ${Ġ}_{0}(t)>0$, *it is interesting to know what happens when* ${Ġ}_{0}(t)=0$. *Theorem* *4* *states that if* $G_{0}(\cdot )$ *has* k *th derivative* $G_{0}^{\left(k\right)}(t)\neq 0$ *and* $G_{0}^{\left(m\right)}(t)=0$ *for* m=1,…k−1, *then* Ĝ(t) *has convergence rate* $I^{k/(2k+1)}$ *faster than the rate* $I^{1/3}$ *in Theorem* *3*. *This result is new for this type of estimator*.

Theorem 4 *Assume conditions of Theorem* *3*, *with the condition* ${Ġ}_{0}(t)>0$ *replaced by* $G_{0}^{\left(j\right)}(t)=0$ *for* j=1,…,k−1, *and* $G_{0}^{\left(k\right)}(t)\neq 0$. *Then for fixed* J, *as* I→∞,

$$I^{k/(2k+1)}\left(Ĝ(t)-G_{0}(t)\right)\overset{D}{\to }Z,$$

*where the distribution function of Z is given by*, ∀x∈R,

$$\begin{array}{cc} P(Z\leq x) & =P\left(\underset{h}{arg min}\left\{\eta (t)D_{2}^{1/2}(t)f{\left(t\right)}^{1/2}𝔹(h)\right.\right.\\ & \;\left.\left.-xD_{1}(t)f(t)h+D_{1}(t)f(t)G_{0}^{\left(k\right)}(t)h^{k+1}/(k+1)!\right\}\geq 0\right), \end{array}$$

*where* $\eta (t),D_{1}(t)$ *and* $D_{2}(t)$ *are given in Theorem* *3*.

The distribution of ${\text{arg min}}_{h\in R}\{𝔹(h)+h^{2}\}$ is called the Chernoff distribution; its distribution and density function are first derived in Groeneboom [21]. Both have no closed‐form and are not easy to evaluate. Kosorok [22] proposed a sampling method for evaluating this distribution. Proof of theorems is left in the Appendix S1.

## 5 Hypothesis Testing

In the surveillance problem to investigate safety signals in the I×J AE‐drug combinations, let $\lambda_{ij}=E(y_{ij})$. Our interest is to test the null hypothesis $H_{0}:y_{ij}$ is not a signal under Poisson. (i=1,…,I;j=1,…,J) vs $H_{1}$: Some of $y_{ij}$ are signals. Recall $\delta_{ij}$ is a latent variable indicating if the zero‐count is from the Poisson distribution or not, and ${\hat{\delta }}_{ij}$ is the conditional expectation of $\delta_{ij}$ given in the EM algorithm in §3.

For a given ith AE, denote $x_{i}={\left(x_{i1},\ldots ,x_{iJ}\right)}^{⊺}$, $y_{i}={\left(y_{i1},\ldots ,y_{iJ}\right)}^{⊺}$ and ${\hat{\delta }}_{i}={\left({\hat{\delta }}_{i1},\ldots ,{\hat{\delta }}_{iJ}\right)}^{⊺}$, by (1) and (2), conditioning on $\left(t_{i},x_{i}\right)$, without loss of generality, we assume the first $v_{i}$ cells are non‐zeros for a fixed i, under null hypothesis, the mass function $y_{i}$ is

$$\begin{array}{ll} p(y_{i}|t_{i},k_{j},x,{\hat{\delta }}_{j};\hat{\beta },Ĝ) & =\left(\overset{J}{\underset{j=1}{\prod }}\frac{{\left(Ĝ(t_{i})\exp({\hat{\beta }}^{⊺}x_{i})\right)}^{y_{ij}}}{y_{ij}!}\right)\\ & \;\times \exp\left\{-\left[\overset{k_{i}}{\underset{j=1}{\sum }}Ĝ(t_{i})\exp({\hat{\beta }}^{⊺}x_{ij})\right.\right.\\ & \;\left.\left.+\overset{J}{\underset{j=k_{i}+1}{\sum }}Ĝ(t_{i}){\hat{\delta }}_{ij}\exp({\hat{\beta }}^{⊺}x_{ij})\right]\right\}, \end{array}$$

$C=\exp\left\{-\left[\sum_{j=1}^{k_{i}}Ĝ(t_{i})\exp({\hat{\beta }}^{⊺}x_{ij})+\sum_{j=k_{i}+1}^{J}Ĝ(b_{i}){\hat{\delta }}_{ij}\exp({\hat{\beta }}^{⊺}x_{ij})\right]\right\}$ is a constant, and

$$p(y_{i}|t_{i},k_{j},x,{\hat{\delta }}_{j};\hat{\beta },Ĝ)\propto \left(\overset{J}{\underset{j=1}{\prod }}\frac{{\left(Ĝ(t_{i})\exp({\hat{\beta }}^{⊺}x_{i})\right)}^{y_{ij}}}{y_{ij}!}\right).$$

Thus, under the null hypothesis, conditioning on $\left(t_{i},x_{i},\hat{\beta },Ĝ(t_{i}),{\hat{\delta }}_{i}\right)$,

$$y_{ij}∼Poisson(\lambda_{ij}),\;\;\;\;\lambda_{ij}=Ĝ(t_{i})\exp({\hat{\beta }}^{⊺}x_{ij})$$

and for given significance level α, the (1−α)th upper quantile Q(1−α) can be easily obtained. If $y_{ij}>Q(1-\alpha )$ for some (i,j), then $H_{0}$ is rejected, and $AE_{i}$ is a signal on drug j.

Note that $H_{0}$ can be rewritten as $H_{0}=\cap_{i,j}^{I,J}H_{0ij}$, where $H_{0ij}$: $y_{ij}$ is not a signal under Poisson for AE i – drug j combination. Various approaches for multiple comparison and significance level correction have been shown to improve the original Bonferroni correction, for example, the Benjamini–Hochberg's correction [23] and the Holm–Bonferroni approach [24]. The former is better for controlling the false discovery rate (FDR); the latter is better for controlling the family‐wise error rate (FWER). We adopt the Holm–Bonferroni approach to control and decrease the number of false‐positive signals because of its adaptive nature. It is a sequential rejective step‐down Bonferroni test, which controls the FWER in a strong sense with greater power than the Bonferroni test.

## 6 Simulation Studies: Finite Sample Properties

We conduct extensive simulation studies under various cases and scenarios to evaluate the finite sample properties of the statistical model and the performance of the proposed signal detection method. We also evaluate the associated parameter estimation in surveillance datasets and the robustness to proportions of true zeros and patterns of background noise in the Appendix S1. Datasets are generated with combinations of drug numbers J, AE types I, and proportions of zeros ρ to mimic datasets from the VigiBase database. Below, we present the details of simulation settings and the results of analyzing the simulated data in terms of estimation and signal detection.

### 6.1 Simulation Settings

**FIGURE 1:**
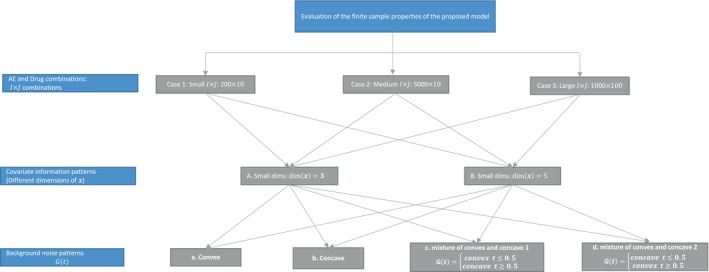
Flow chart of all the simulation scenarios: we explore various scenarios by setting various parameters of sample size I×J, signal strength ρ, various covariate information patterns x, and background noise patterns G(t).

We consider different combinations of I, J, and ρ to reflect the VigiBase datasets. Also, we evaluate the accuracy and precision of the estimates in different types of potential covariate information by setting different dimensions of x. Moreover, we evaluate different background noise patterns by setting different shapes of G(t). Design details of each simulation scenario, of sample size N=1000, are as follows. Figure 1 shows the scenarios that we consider in this paper.

Case 1: I=200,J=10,ρ∼uniform(0.3,0.5).

x is sampled from a 3‐dimensional multivariate normal distribution, then scenarios 1–4:

Scenario 1: G(t) is a convex function.
Scenario 2: G(t) is a concave function.
Scenario 3: G(t) is convex when t≤0.5 and G(t) is concave when t≥0.5.
Scenario 4: G(t) is concave when t≤0.5 and G(t) is convex when t≥0.5.
  Denote $x={\left(x_{1},x_{2},x_{3}\right)}^{⊺}$, $x_{1}$ is sampled from a 2‐dimensional multivariate normal distribution, $x_{2}$ is sample from a binomial distribution, and $x_{3}$ is randomly sampled from (1,2,3,4,5,6), then scenarios 5–8:
Scenario 5: G(t) is a convex function.
Scenario 6: G(t) is a concave function.
Scenario 7: G(t) is convex when t≤0.5 and G(t) is concave when t≥0.5.
Scenario 8: G(t) is concave when t≤0.5 and G(t) is convex when t≥0.5.
x is sampled from a 5‐dimensional multivariate normal distribution, then scenarios 9–12:
Scenario 9: G(t) is a convex function.
Scenario 10: G(t) is a concave function.
Scenario 11: G(t) is convex when t≤0.5 and G(t) is concave when t≥0.5.
Scenario 12: G(t) is concave when t≤0.5 and G(t) is convex when t≥0.5.

Case 2: I=5000,J=10,ρ∼uniform(0.2,0.4). Scenarios 1–12 are the same as case 1.

Case 3: I=1000,J=100,ρ∼uniform(0.3,0.6). Scenarios 1–12 are the same as case 1.

In the data generation process, we generate a true zero indicator $z_{ij}$ from a binomial distribution. If $z_{ij}$ is equal to 1, we set $y_{ij}$ to be 0. Otherwise, we generate $y_{ij}$ from a Poisson distribution with a conditional mean of $E(y_{ij}(t)|x_{j})=rr_{ij}G(t_{i})\exp(\beta^{⊺}x_{ij})$. If the AE i on drug j is sampled as a signal, then $rr_{ij}=1.5$ (or 2,3,4,6), if not, $rr_{ij}=1$. We use different rr to represent AE signals with different strengths. We set different true numbers of signals at 0 (null hypothesis), 50, 100, 350, and 700.

### 6.2 Performance Evaluation Measures

We evaluate the proposed method's parameter‐estimation accuracy using bias, defined as the average difference between the estimator and the true value. We consider the proposed method's precision by standard deviation. Also, we evaluate the proposed method's performance in signal detection by Type I error/Power, False Discovery Rate (FDR), Specificity, and Sensitivity.

#### 6.2.1 Type I Error/Power

We assume the null hypothesis is rejected if at least one signal is found in a simulated dataset. Then, a type I error is defined as the number of times the null hypothesis is rejected in the N simulated datasets under the null hypothesis. Power is defined as the proportion of times the null hypothesis is rejected in N simulated datasets under the alternative hypothesis.

#### 6.2.2 False Discovery Rate (FDR)

FDR is defined as the expected proportion of false‐positive signals among all signals found. It is a common measure in multiple comparisons or search analysis, and is estimated as

$$FDR=\overset{N}{\underset{j=1}{\sum }}\frac{\text{number of false signals found in simulation j}}{\text{number of all signals found in simulation j}}/N.$$

#### 6.2.3 Specificity

Specificity measures the proportion of negatives that are correctly identified, and is estimated as

$$Spe=\overset{N}{\underset{j=1}{\sum }}\frac{\text{number of true non‐signals found in simulation j}}{\text{number of all non‐signals in simulation j}}/N.$$

#### 6.2.4 Sensitivity

Sensitivity measures the proportion of positives that are correctly identified, and is estimated by

$$Sen=\overset{N}{\underset{j=1}{\sum }}\frac{\text{number of true signals found in simulation j}}{\text{number of true signals in simulation j}}/N.$$

We compare the results from PRR, BCPNN, ZIP LRT, and the proposed approach, with and without incorporating covariates information (NHP) in the panel with signals generated with a higher‐than‐average conditional mean, which are shown in the Appendix S1.

### 6.3 Simulation Results

#### 6.3.1 Simulation Results: Signal Detection

The simulation findings (Table 1) confirm that the type I error is around 0.05 under the null hypothesis, demonstrating that Holm‐Bonferroni correction controls the type I error very well. Under the alternative hypothesis, the power for the proposed approach is close to 100%. Within Case 2 (1000×100), and when the number of true signals is 100, as the signal becomes strong, Table 1 shows that the specificity of the proposed method decreases from >99.99% to 99.98%, the sensitivity increases from 93.23% to >99.99%, and FDR increases from <0.01 to 0.009. As the number of true signals increases from 100 to 1000, Sensitivity and Specificity do not change much, and FDR decreases. Similar conclusions hold for other cases (200×10 and 5000×10) and within each case. As the sample sizes increase from 200×10 to 1000×100, Specificity increases, Sensitivity and FDR decrease. In general, the proposed approach has high power (>99.99%), high sensitivity (92.78% ‐), and high specificity (91.48% ‐). As the signal strength increases, the specificity decreases, and the sensitivity increases.

**TABLE 1:** Performance characteristic of the proposed method in signal detection when signal strength (rr) or sample size varies.

True #	rr	Power(%)	Specificity(%)	Sensitivity(%)	FDR
I=200,J=10					
0	—	0.060^a^	—	—	—
50	1.5	>99.99	99.79	95.40	0.033
50	2	>99.99	99.39	99.66	0.071
50	4	>99.99	93.53	>99.99	0.327
50	6	>99.99	91.48	>99.99	0.382
100	1.5	>99.99	95.91	99.92	0.009
100	2	>99.99	99.47	99.54	0.038
100	4	>99.99	96.49	>99.99	0.151
100	6	>99.99	94.88	>99.99	0.224
350	1.5	>99.99	99.95	93.06	0.001
350	2	>99.99	99.86	99.21	0.004
350	4	>99.99	99.24	99.97	0.014
350	6	>99.99	99.61	99.99	0.009
I=1000, J=100					
0	—	0.02	—	—	—
100	1.5	>99.99	>99.99	93.23	<0.001
100	2	>99.99	>99.99	99.08	<0.001
100	4	>99.99	99.93	99.98	0.021
100	6	>99.99	99.98	>99.99	0.037
350	1.5	>99.99	>99.99	92.91	<0.001
350	2	>99.99	>99.99	99.22	<0.001
350	4	>99.99	99.71	99.99	0.015
350	6	>99.99	99.99	>99.99	0.009
700	1.5	>99.99	99.99	93.12	<0.001
700	2	>99.99	>99.99	99.26	0.003
700	4	>99.99	99.98	>99.99	0.006
700	6	>99.99	99.42	>99.99	0.059
1000	1.5	>99.99	>99.99	92.78	<0.001
1000	2	>99.99	>99.99	99.14	<0.001
1000	4	>99.99	99.90	99.99	0.013
1000	6	>99.99	99.57	>99.99	0.024
I=5000, J=10					
0	—	0.04	—	—	—
100	1.5	>99.99	>99.99	93.54	0.001
100	2	>99.99	99.94	99.37	0.011
100	4	>99.99	99.38	99.97	0.040
100	6	>99.99	98.98	>99.99	0.048
600	1.5	>99.99	99.99	93.86	0.004
600	2	>99.99	99.92	99.11	0.013
600	4	>99.99	97.96	99.99	0.057
600	6	>99.99	98.98	>99.99	0.073
1000	1.5	>99.99	>99.99	93.50	<0.001
1000	2	>99.99	99.68	99.26	0.016
1000	4	>99.99	98.09	99.99	0.056
1000	6	>99.99	92.25	92.87	0.184

^a^ When the simulated data are under null hypothesis, this column indicates type I error.

#### 6.3.2 Simulation Results From PRR, BCPNN, ZIP LRT

The simulated data are generated with covariate information. Tables 2 and 3 show the performance characteristic of these methods without covariates information. Table 2 shows the performance metrics of the proposed method when setting the covariates as **1**, in other words, when ignoring the covariates information. Within Case 2 (1000×100), the power is close to 1. When the number of true signals is 1000, as the signal strength increases, the specificity increases from 74.95% to 76.36%, the sensitivity increases from 32.59% to 57.35%, and the FDR decreases from 0.976 to 0.957. A similar trend holds for 100, 350, and 700 signals. Specificity, Sensitivity, and FDR do not change as the number of signals changes. Similar conclusions hold for other sample size settings (200×10 and 5000×10). In general, NHP has medium specificity (73.51%–88.83%) among all these approaches without covariates, but it also has low sensitivity (32.87%–62.18%).

**TABLE 2:** Performance characteristic of the proposed method without covariates information in signal detection when signal strength (rr) or sample size varies.

True #	rr	Power(%)	Specificity(%)	Sensitivity (%)	FDR
I=200,J=10					
0	—	0.060	—	—	0.060
50	1.5	>99.99	74.01	35.94	0.943
50	2	>99.99	73.71	40.94	0.936
50	4	>99.99	75.74	52.28	0.914
50	6	>99.99	77.44	59.06	0.897
100	1.5	>99.99	73.65	35.20	0.892
100	2	>99.99	75.01	39.72	0.873
100	4	>99.99	77.92	51.21	0.825
100	6	>99.99	80.07	55.76	0.796
350	1.5	>99.99	75.77	32.42	0.646
350	2	>99.99	78.58	36.16	0.591
350	4	>99.99	84.92	42.96	0.462
350	6	>99.99	88.83	46.77	0.371
I=1000,J=100					
0	—	0.02	—	—	0.02
100	1.5	>99.99	75.07	32.87	0.998
100	2	>99.99	74.86	38.51	0.997
100	4	>99.99	74.89	50.46	0.996
100	6	>99.99	75.07	59.49	0.996
350	1.5	>99.99	75.10	32.87	0.992
350	2	>99.99	74.84	38.47	0.990
350	4	>99.99	74.92	52.26	0.987
350	6	>99.99	75.40	58.26	0.985
700	1.5	>99.99	74.91	32.87	0.983
700	2	>99.99	75.22	37.48	0.981
700	4	>99.99	75.59	50.90	0.974
700	6	>99.99	75.89	57.82	0.970
1000	1.5	>99.99	74.95	32.59	0.976
1000	2	>99.99	75.04	37.93	0.973
1000	4	>99.99	75.87	50.46	0.963
1000	6	>99.99	76.36	57.85	0.957
I=5000,J=10					
0	—	0.04	—	—	0.04
100	1.5	>99.99	74.09	34.17	0.996
100	2	>99.99	73.98	39.64	0.996
100	4	>99.99	73.51	53.21	0.994
100	6	>99.99	74.53	62.18	0.993
600	1.5	>99.99	74.03	34.47	0.977
600	2	>99.99	74.19	40.04	0.974
600	4	>99.99	74.70	52.59	0.965
600	6	>99.99	75.58	61.96	0.957
1000	1.5	>99.99	74.48	33.95	0.962
1000	2	>99.99	74.42	39.99	0.956
1000	4	>99.99	75.39	53.10	0.940
1000	6	>99.99	76.88	59.49	0.929

**TABLE 3:** Performance characteristic of PRR, BCPNN, and ZIP LRT in signal detection when signal strength (rr) or sample size varies.

		PRR				BCPNN				ZIPLRT			
True #	rr	Power(%)	Specificity(%)	Sensitivity(%)	FDR	Power(%)	Specificity(%)	Sensitivity(%)	FDR	Power(%)	Specificity(%)	Sensitivity(%)	FDR
I=200,J=10													
0	—	1	—	—	1	1	—	—	1	1	—	—	1
50	1.5	>99.99	60.57	80.46	0.950	>99.99	59.35	81.78	0.951	>99.99	63.34	36.14	0.975
50	2	>99.99	60.95	86.38	0.946	>99.99	59.76	87.30	0.947	>99.99	63.35	36.10	0.975
50	4	>99.99	61.91	95.88	0.939	>99.99	60.73	96.12	0.941	>99.99	64.19	35.02	0.976
50	6	>99.99	62.57	97.38	0.937	>99.99	61.51	97.62	0.939	>99.99	64.45	36.34	0.974
100	1.5	>99.99	61.70	78.36	0.903	>99.99	60.51	79.83	0.904	>99.99	63.52	37.25	0.949
100	2	>99.99	62.01	85.67	0.894	>99.99	60.83	86.80	0.896	>99.99	63.55	36.46	0.950
100	4	>99.99	63.91	93.98	0.879	>99.99	62.79	94.35	0.882	>99.99	64.70	35.87	0.949
100	6	>99.99	65.37	96.37	0.872	>99.99	64.28	96.60	0.875	>99.99	65.61	33.87	0.951
350	1.5	>99.99	67.58	77.01	0.665	>99.99	66.60	78.37	0.668	>99.99	63.36	36.44	0.826
350	2	>99.99	69.30	81.75	0.639	>99.99	68.28	82.75	0.644	>99.99	64.09	36.09	0.824
350	4	>99.99	74.23	88.27	0.579	>99.99	73.42	88.86	0.585	>99.99	66.57	33.71	0.824
350	6	>99.99	77.34	90.25	0.542	>99.99	76.61	90.59	0.549	>99.99	68.12	32.02	0.824
I=1000,J=100													
0	—	1	—	—	1	1	—	—	1	1	—	—	1
100	1.5	>99.99	63.27	82.53	0.998	>99.99	62.00	84.15	0.998	>99.99	67.56	31.93	0.999
100	2	>99.99	63.32	89.67	0.998	>99.99	62.00	90.65	0.998	>99.99	67.55	32.36	0.999
100	4	>99.99	63.39	98.05	0.997	>99.99	62.11	98.36	0.997	>99.99	67.64	32.59	0.999
100	6	>99.99	63.53	99.56	0.997	>99.99	62.18	99.63	0.997	>99.99	67.70	31.68	0.999
350	1.5	>99.99	63.42	82.39	0.992	>99.99	62.14	84.04	0.992	>99.99	67.55	32.41	0.997
350	2	>99.99	63.55	90.03	0.991	>99.99	62.21	91.11	0.992	>99.99	67.59	33.16	0.996
350	4	>99.99	63.79	98.17	0.991	>99.99	62.49	98.42	0.991	>99.99	67.85	32.14	0.997
350	6	>99.99	64.07	99.48	0.990	>99.99	62.74	99.57	0.991	>99.99	68.10	32.06	0.996
700	1.5	>99.99	63.57	82.14	0.984	>99.99	62.26	83.74	0.985	>99.99	67.60	32.77	0.993
700	2	>99.99	63.70	89.77	0.983	>99.99	62.45	90.85	0.983	>99.99	67.75	32.37	0.993
700	4	>99.99	64.34	98.11	0.981	>99.99	63.06	98.37	0.982	>99.99	68.12	31.84	0.993
700	6	>99.99	64.87	99.39	0.980	>99.99	63.55	99.47	0.981	>99.99	68.60	31.30	0.993
1000	1.5	>99.99	63.74	82.13	0.978	>99.99	62.44	83.72	0.978	>99.99	67.59	32.66	0.990
1000	2	>99.99	63.94	89.53	0.976	>99.99	62.68	90.60	0.976	>99.99	67.72	32.38	0.990
1000	4	>99.99	64.86	98.03	0.973	>99.99	63.52	98.29	0.974	>99.99	68.31	31.91	0.990
1000	6	>99.99	65.49	99.36	0.972	>99.99	64.24	99.44	0.973	>99.99	68.99	31.31	0.990
I=5000,J=10													
0	—	1	—	—	1	1	—	—	1	1	—	—	1
100	1.5	>99.99	57.50	74.94	0.749	>99.99	56.12	76.43	0.997	>99.99	63.01	36.94	0.998
100	2	>99.99	57.57	84.13	0.996	>99.99	56.15	85.07	0.996	>99.99	62.98	37.04	0.998
100	4	>99.99	57.79	95.49	0.995	>99.99	56.33	95.84	0.996	>99.99	62.81	37.65	0.998
100	6	>99.99	57.81	98.06	0.995	>99.99	56.47	98.27	0.995	>99.99	63.17	36.89	0.998
600	1.5	>99.99	58.03	75.19	0.979	>99.99	56.62	76.70	0.979	>99.99	62.99	37.04	0.988
600	2	>99.99	58.10	83.25	0.976	>99.99	56.75	84.37	0.977	>99.99	62.85	37.12	0.988
600	4	>99.99	58.69	95.12	0.973	>99.99	57.34	95.48	0.974	>99.99	63.18	36.83	0.988
600	6	>99.99	58.96	97.77	0.972	>99.99	57.61	97.93	0.973	>99.99	63.51	36.45	0.988
1000	1.5	>99.99	58.38	75.01	0.965	>99.99	57.02	76.53	0.965	>99.99	63.02	37.20	0.980
1000	2	>99.99	58.46	83.13	0.961	>99.99	57.07	84.28	0.961	>99.99	62.94	37.06	0.980
1000	4	>99.99	59.30	94.55	0.955	>99.99	57.93	94.95	0.956	>99.99	63.40	36.35	0.980
1000	6	>99.99	59.83	97.37	0.953	>99.99	58.51	97.56	0.954	>99.99	64.06	35.76	0.980

Table 3 shows the performance metrics of the classic methods (PRR, BCPNN, and ZIPLRT) when ignoring the covariates information. Within Case 2 (1000×100), the power of PRR is close to 1. When the number of true signals is 1000, as the signal strength increases, the specificity increases from 63.74% to 65.49%, the sensitivity increases from 82.13% to 99.36%, and the FDR is around 0.976. A similar trend holds for 100, 350, and 700 signals. Specificity, Sensitivity, and FDR do not change much as the number of signals changes. Similar conclusions hold for other sample size settings (200×10 and 5000×10). In general, PRR has high sensitivity (74.94%–99.48%) but low specificity (57.50%–77.34%). Within Case 2 (1000×100), the power of BCPNN is close to 1. When the number of true signals is 1000, as the signal strength increases, the specificity increases from 62.44% to 65.49%, the sensitivity increases from 83.72% to 99.44%, and the FDR is around 0.976. A similar trend holds for 100, 350, and 700 signals. Specificity, Sensitivity, and FDR do not change much as the number of signals changes. Similar conclusions hold for other sample size settings (200×10 and 5000×10). In general, BCPNN is similar to PRR, with high sensitivity (78.37%–99.63%) and low specificity (56.12%–76.61%). Within Case 2 (1000×100), the power of ZIP LRT is close to 1. When the number of true signals is 1000, as the signal strength increases, the specificity increases from 67.59% to 68.99%, the sensitivity decreases from 32.66% to 31.31%, and the FDR is around 0.990. A similar trend holds for 100, 350, and 700 signals. Specificity, Sensitivity, and FDR do not change much as the number of signals changes. Similar conclusions hold for other sample size settings (200×10 and 5000×10). In general, ZIP LRT has low specificity (62.81%–68.99%) and low sensitivity (31.30%–37.65%).

Table 4 shows the number of signals found with different approaches. Those without incorporating covariates information (PRR, BCPNN, ZIP LRT, NHP) perform pretty poorly and tend to heavily overestimate the number of signals, except NHP, which may over‐ or underestimate. In contrast, the proposed approach with covariates adjustment finds the true number of signals with outstanding accuracy, demonstrating the additional value of the proposed method over the existing ones.

**TABLE 4:** Signals (AEs) detected by the proposed method, the proposed method without covariates, PRR, BCPNN, and ZIPLRT.

True #	rr	PRR	BCPNN	ZIPLRT	NHP	HP
I=200,J=10						
0	—	813	836	734	321	0.07
50	1.5	809	834	733	317	50
50	2	805	827	730	321	57
50	4	794	816	721	307	124
350	1.5	804	825	732	320	326
350	2	789	810	721	309	348
350	4	737	753	673	279	356
I=1000,J=100						
0	—	36774	38075	32472	13879	0.02
350	1.5	36744	38022	32446	13697	325
350	2	36726	37960	32212	13888	347
350	8	35911	37221	31737	13589	545
1000	1.5	36719	38018	32415	13833	928
1000	2	36577	37855	32312	13885	991
1000	8	34425	35720	30352	13023	1320
I=5000,J=10						
0	—	21302	22014	18587	9119	0.04
100	1.5	21282	21970	18494	9106	94
100	3	21248	21937	18555	8972	102
100	6	21151	21821	18413	8940	467
600	1.5	21185	21888	18507	9162	567
600	4	20976	21647	18411	9005	1306
600	6	20860	21527	18245	8789	953

### 6.4 Convergence and Robustness Across Initialization Scenarios

To assess sensitivity, we conducted supplementary analyses using multiple, widely dispersed starting values for $\beta^{\left(0\right)}$. Across these runs, the algorithm consistently converged to the true value of β (within numerical tolerance), and the resulting parameter estimates and fit metrics remained effectively unchanged.

We further evaluated sensitivity through six distinct simulation scenarios (see Appendix S1) designed to examine the dependence of the EM‐isotonic procedure on initialization. These scenarios demonstrate that the choice of starting values does not impact the model's final convergence (within numerical tolerance).

As an illustration, consider the scenario with I=200,J=10, a concave true G(·), and true parameter value β=(1.2,2.9,0.6). In this case, the algorithm consistently converged to the same solution across independent runs, with each scenario repeated 1000 times. We summarize all simulation results in Appendix S1: estimates of (β,G(·)) remain stable as $\left(\beta^{\left(0\right)},G^{\left(0\right)}\right)$ are varied. The bias and standard deviation show no evident trend across initialization scenarios.

## 7 Application to WHO VigiBase

We apply the proposed model to the WHO VigiBase for cases submitted to the WHO International Drug Monitoring System between January 2000 to June 2020. The VigiBase reports include patient demographics, reported drugs, and events. We apply the reported drugs we are interested in, common cardiovascular medicines, in this analysis, as column names of our dataset. Furthermore, the reported events have been coded according to Medical Dictionary for Regulatory Activities (MedDRA). Therefore, we use the highest level in MedDRA, System Organ Class (SOC), to identify the categories of AEs we plan to investigate. And we use a lower‐level term, Preferred Terms (PT), in MedDRA to define thesingle AE as the row name in the analysis, such as malaise, hepatitis, and nervousness. Moreover, patient demographics, including age, gender, and country, are used as covariates.

In the VigiBase dataset, we investigate the association between some common cardiovascular medicines and corresponding AEs. We will perform signal detection in this database and discuss the public health implications from comparisons with other approaches, including PRR, BCPNN, and ZIP LRT, which do not incorporate covariates. The structure of the datasets may differ for different investigative questions.

### 7.1 Covariates Selection and Data Preparation

Vascular disorder‐related AEs are very common among patients who are prescribed cardiovascular medicines. In this case, we want to detect vascular disorder‐related AEs signals associated with Acebutolol, Apixaban, and other eight cardiovascular medicines used to treat thrombosis. We extract all the vascular disorder‐related AEs, such as hematoma, hemorrhage, and hyperemia, against one of the 10 medicines reported during the analysis period. After data processing, there are 202 vascular disorder‐related AEs against at least one of 10 drugs. After collapsing the data into the form of 202×10 table, ρ=68.6% of the cells are zeros; for example, no occurrence of angiopathy AE is reported against drug Amiodarone, then the cell count of angiopathy‐Amiodarone is zero. The total number of occurrences of AEs is $y_{\cdot \cdot }=30488$. AE hypotension has the highest occurrence, which is 6513 in total ($\max(y_{i\cdot })=6513$), and AE aortic dissection rupture has the lowest occurrence, which is $y_{adr\cot}=1$. We let $t_{i}=y_{i\cdot }/\max(y_{i\cdot })$ to adjust for background noise $G(t_{i})$ for AE i. For example, $t_{i}=1/6513$ for AE hypotension. In particular, Theorem 3 holds as stated for any fixed t, but it does not establish uniform validity over the entire range of t. Accordingly, when using $Ĝ(t_{i})$ to estimate $G(t_{i})$, the interpretation is local in t: for each given $t_{i}$, $Ĝ(t_{i})$ is intended to provide an accurate estimate of $G(t_{i})$ at that specific index value, rather than uniformly over all $t_{i}$, i=1,…,I. This also means that, in the downstream application, the model‐based estimate should be evaluated separately for each index value $t_{i}$ when making signal‐detection decisions.

It is worth noting that age and gender are very important factors that affect the occurrence of AEs [25, 26]. In addition, geographical variation is also a crucial factor that affects the occurrence of AEs, since the medical service and emphasis on different kinds of AEs vary in different countries [27]. Also, we note that patients' age, gender, and country vary across drugs in our data. For example, the average age for Amfetamine users is 35.25, and the average age for Apixaban users is 74.87 in this dataset. Moreover, the proportion of female users for Amiodarone is 0.41, whereas it is 0.76 for Ambrisentan users. So, it is crucial to adjust for confounders in the signal detection process to remove imbalances arising from patient characteristics such as age, gender, and country.

### 7.2 Signal Detection With the Proposed Model and Existing Methods

The results show that if we do not incorporate covariates information, PRR finds 229 signals; BCPNN finds 148 signals; the proposed approach without covariates (NHP) finds 57 signals, and ZIP LRT finds 62 signals. When we incorporate information such as country, gender, and age, the proposed approach finds 89 signals. We compare our results with those signals detected by those without incorporating demographic information.

**FIGURE 2:**
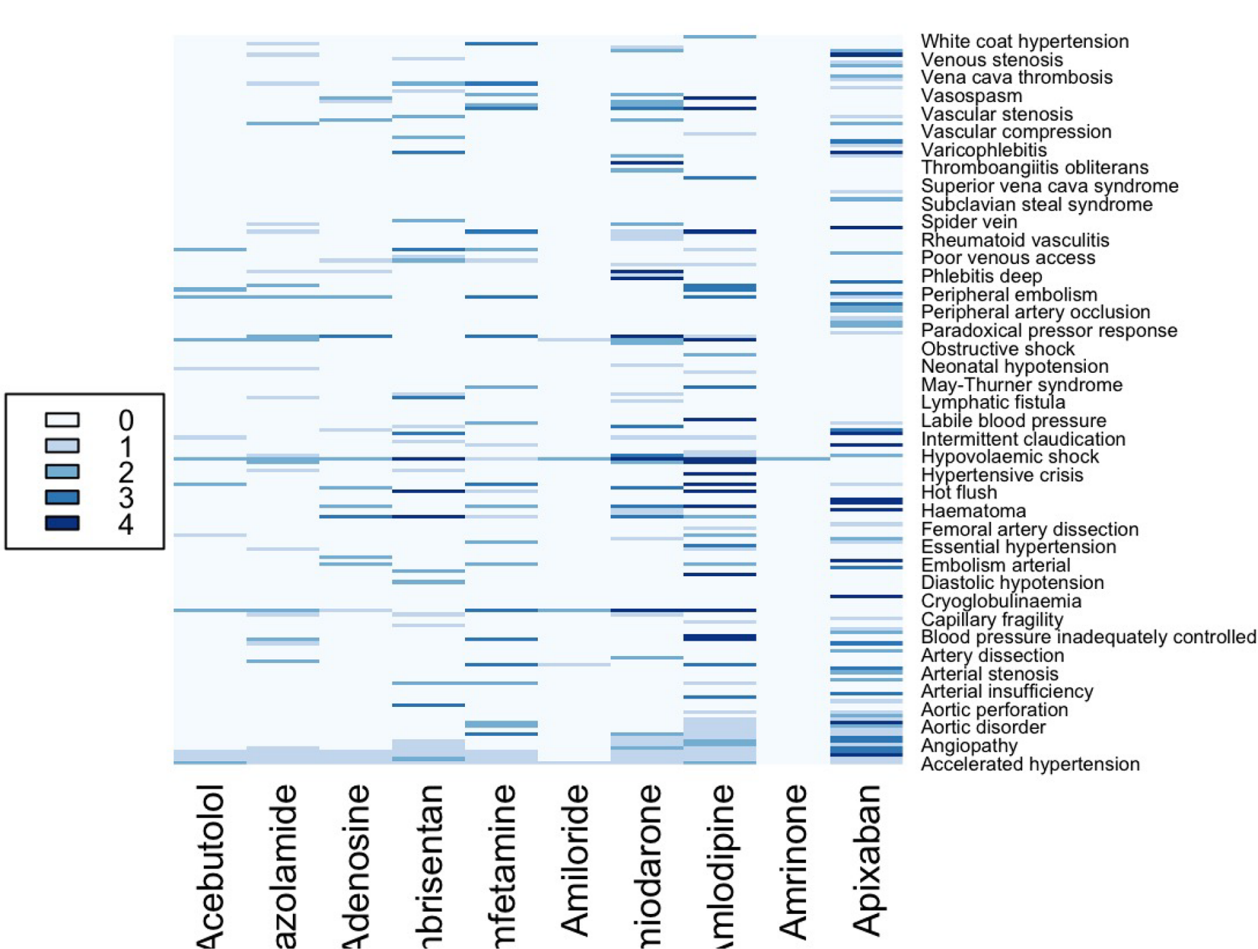
Visualization of the signals detected by PRR, BCPNN, ZIPLRT, and the proposed methods with and without incorporating covariates information: the deeper the color is, the more frequent the signal has been detected by the methods.

Figure 2 describes the signals detected by PRR, BCPNN, ZIPLRT, NPH, and the proposed method, which shows that the combination of all the methods has displayed that most of the AEs in the study were associated of substance Apixaban, and almost no AE was associated of Amrinone. One of the possibilities is that more patients have been prescribed Apixaban.

Because the sensitivity of all the other approaches is lower than the proposed one with covariates information, PRR/BCPNN/ZIP LRT/NHP might miss some true positive signals. LRT misses 87 of the 89 signals detected by the proposed approach; PRR misses 14; and BCPNN misses 29. Even the PRR, BCPNN, and ZIP LRT combined, still 11 signals are missed among the 89 signals detected by the proposed approach. For example, 809 flushing cases are reported for the drug Amlodipine; the PRR of the flushing AE on the drug and its 95% CI are 0.161 (0.012,0.309), whose lower bound is smaller than 1. However, after incorporating covariates such as gender based on the average level rather than PRR, the proposed approach detected it as a signal. We prefer to recognize this as a true signal because the proposed approach has a higher specificity and sensitivity than others do. However, two signals are found by all other approaches (PRR, BCPNN, NHP, ZIP LRT) but are missed by the proposed approach. The signals found by all the other methods other than the proposed method are Aneurysm on Apixaban and Aortic dissection on Apixaban. The occurrence of AEs for the two signals is 43 and 18, respectively. Since PRR/BCPNN/NHP/ZIP LRTs do not control for type I error and FDR well when accounting for covariates, the two signals might be false positives.

## 8 Conclusion and Discussion

We propose a general semiparametric panel count model for inferring associations between adverse events (AEs) and drugs in post‐market surveillance systems, where enriched drug safety information and patient‐level data are available. A nonhomogeneous process models the cell counts for the I×J AE‐drug combinations. The nonparametric component G(·) of the model adjusts for the background occurrence of AE, while the parametric component incorporates covariates information while accounting for excessive zeros via a latent variable. Signals are detected by comparing AE‐drug combinations of interest with their expected values, thereby avoiding the bias common to RR‐based approaches [6]. The proposed model is flexible and robust to modeling assumptions and handles excess zeros more appropriately than the ZIP model, a longstanding concern in signal detection for surveillance systems. To our knowledge, this is the first work to jointly incorporate covariate information while explicitly addressing excess zeros in these systems.

We further show that hypothesis testing based on the proposed estimates is effective for signal detection while accounting for the joint effect of many AEs. For example, LRT‐based approaches [8] rely on Monte Carlo simulation to obtain empirical null distributions of the test statistics, which is time‐consuming. Joint analysis enables assessment of AEs' collective roles for each drug. Simulations demonstrate high power and sensitivity with high specificity. As discussed in §6, existing approaches such as PRR, BCPNN, and more recent ZIP‐based LRT can miss important signals. Importantly, the ability to adjust for covariates provides a powerful avenue for analyses in the evolving FDA Sentinel system, which is being expanded to collect richer information per the Sentinel System 5‐Year Strategy.

For multiple testing, we adopt the Holm‐Bonferroni procedure. The Holm‐Bonferroni method is a step‐down refinement of Bonferroni: it sequentially adjusts the significance level based on the number of remaining hypotheses, making it less conservative than the traditional Bonferroni correction while still providing strong familywise error rate control. This step‐down approach typically achieves a better balance between identifying true effects and limiting false positives. If a drug's safety profile is well established and more conservative monitoring is warranted, the Bonferroni correction may be appropriate, though it typically yields fewer signals. Alternatively, false discovery rate (FDR) control methods such as Benjamini–Hochberg [23] can be used; they control FDR (and imply weaker FWER control) but generally rely on independence assumptions that may be questionable in this context.

On the other hand, the proposed method still shares several limitations with existing approaches. When many potential signals occur within the same panel, the expected number of signals increases, which can raise the risk of false negatives. In addition, the correlation among AEs is currently ignored. Incorporating a general dependence structure across thousands of AEs would require very high‐dimensional modeling and is computationally prohibitive for large‐scale surveillance. Such dependence could, in principle, be modeled within our framework via shared frailty. We plan to explore these extensions in future work. Incorporating a frailty term to account for AE correlation would require integrating a high‐dimensional likelihood or treating the problem as high‐dimensional missing data, which may be computationally prohibitive and is beyond the scope of the present manuscript.

To compute the nonparametric MLE of the link function G(·), shape constraints are required (e.g., monotonicity, unimodality, convexity/concavity, or log‐convexity/log‐concavity); otherwise, the MLE may not exist in general. The monotonicity may be restrictive for some AE patterns, but it is reasonable in applications; in contrast, the commonly used parametric linear model and logistic model, etc., are also monotonic. We view the convex/concave constraint as relatively relaxed because it encompasses many monotone functions. This topic will be the focus of future study. Also, Theorems 1 and 2 establish the asymptotic properties of the maximum likelihood estimator $\left(\hat{\beta },Ĝ\right)$. In practice, the computation of $\left(\hat{\beta },Ĝ\right)$ involves an E‐step in the EM algorithm. Since an approximation is used in the E‐step, the implemented procedure is more precisely a generalized EM algorithm. According to our simulation studies, this approximation performs well in practice and appears to provide a close numerical approximation to the estimator. More generally, this distinction is not unique to the present setting: even for a standard EM algorithm without approximation in the E‐step, the theoretical results are stated for the true maximum likelihood estimator(MLE) as the number of iterations goes to infinity, whereas the estimator obtained in practice is based on a finite number of iterations and is therefore only a numerical approximation to the true MLE. In our setting, the asymptotic results in Theorems 1 and 2 should likewise be interpreted as applying to the “true” MLE. Based on the simulation studies, the approximation does not appear to materially affect the empirical performance of the procedure.

Finally, the semiparametric panel count model is broadly applicable to other settings with count outcomes, such as numbers of accidents, product defects, and insurance claims. The EM algorithm with an isotonic regression step in each M‐step for parameter estimation that we developed here provides a foundation that can be adapted to related areas. In addition, when richer longitudinal follow‐up information is available, our framework could be extended to incorporate temporal components (e.g., time‐varying intensities or lag structures) and validated against known latency profiles.

## Funding

The open access funding is provided via the Georgetown University‐Wiley institutional agreement.

## Disclosure

Uppsala Monitoring Centre, National Centres (UMC) provided the data extracted from VigiBase, but the information and the study results do not represent the UMC or WHO's opinion. Also, the information comes from various sources, and the suspected adverse effects are drug‐related and not the same in all cases.

## Conflicts of Interest

The authors declare no conflicts of interest.

## Supporting information




**Data S1. Table 1.** Parameter estimation of the proposed model under Cases 1–3 when sample size, dimension of x or shape of G(t) varies. **Table 2.** Performance characteristic of the proposed method when the proportions of true zeros (ρ(%)) and sample size varies. **Table 3.** Performance characteristic of the proposed method when rr is extreme small and the signals are extremeweak. **Table 4.** Convergence and robustness across initialization scenarios: β estimates, bias, and standard deviation **Figure 1.** Estimates of G(t) when G(t) is convex, concave, a mixture of convex and concave, sample size is 200×10 under Case 1: Solid line: True $G_{0}(\cdot )$; Step line: Estimate Ĝ(·). **Figure 2.** Estimates of G(t) when G(t) is convex, concave, a mixture of convex and concave, sample size is 5000 × 10 under Case 2: Solid line: True $G_{0}(\cdot )$; Step line: Estimate Ĝ(·). **Figure 3.** Estimates of G(t) when G(t) is convex, concave, a mixture of convex and concave, sample size is 1000×100 under Case 3: Solid line: True $G_{0}(\cdot )$; Step line: Estimate Ĝ(·). **Figure 4.** Estimated G(·) across six initialization scenarios: Solid line: True $G_{0}(\cdot )$; Step line: Estimate Ĝ(·).

## Data Availability

The authors have nothing to report.
