# Supplementary material for: Semiparametric Panel Count Model, With Applications to Signal Detection in Post‐Market Drug Surveillance Systems
Source: Stat Med. 2026 Jul 9;45(15-17):e70649. doi: 10.1002/sim.70649 (PMC13347285; doi:10.1002/sim.70649)
Supplement: Supplementary file 1 — Data S1. Table 1. Parameter estimation of the proposed model under Cases 1–3 when sample size, dimension of x or shape of G(t) varies. Table 2. Performance characteristic of the proposed method when the proportions of true zeros (ρ(%)) and sample size varies. Table 3. Performance characteristic of the proposed method when rr is extreme small and the signals are extremeweak. Table 4. Convergence and robustness across initialization scenarios: β estimates, bias, and standard deviation Figure 1. Estimates of G(t) when G(t) is convex, concave, a mixture of convex and concave, sample size is 200×10 under Case 1: Solid line: True G0(·); Step line: Estimate Ĝ(·). Figure 2. Estimates of G(t) when G(t) is convex, concave, a mixture of convex and concave, sample size is 5000 × 10 under Case 2: Solid line: True G0(·); Step line: Estimate Ĝ(·). Figure 3. Estimates of G(t) when G(t) is convex, concave, a mixture of convex and concave, sample size is 1000×100 under Case 3: Solid line: True G0(·); Step line: Estimate Ĝ(·). Figure 4. Estimated G(·) across six initialization scenarios: Solid line: True G0(·); Step line: Estimate Ĝ(·). [file SIM-45-0-s001.pdf]

ORIGINAL ARTICLE

Journal Section

Semi-parametric Panel Count Model, with Applications to Signal Detection in Post-market Drug Surveillance Systems

Yizhao Zhou PhD[1*] | Ao Yuan PhD[2*] | Ming Tan PhD[3*]

Supplementary Material

| **Web Appendix A**

proof of Theorem 1 Let $\boldsymbol{s} := (t, \boldsymbol{y}, \boldsymbol{x})$, $\ell(\boldsymbol{\theta}, G|\boldsymbol{s}) = \log p(\boldsymbol{y}|t, \boldsymbol{x}; \boldsymbol{\theta}, G)$ be the log-likelihood of the observed data, with $p(\boldsymbol{y}|t, \boldsymbol{x}; \boldsymbol{\theta}, G)$ given in (2), $d(\boldsymbol{\theta}, \boldsymbol{\theta}_0; G, G_0) = \left(\sum_{j=1}^{d}(\beta_j - \beta_{0,j})^2\right)^{1/2} + |\lambda - \lambda_0| + \sup_{t\in[0,1]} |G(t) - G_0(t)|$, and $(\hat{\boldsymbol{\theta}}, \hat{G})$ be the MLE of $(\boldsymbol{\theta}_0, G_0)$ under the log-likelihood of the observed data. Note that $(\hat{\boldsymbol{\beta}}, \hat{G})$ is the same as that given by the EM-algorithm. In fact we will show more than what claimed:

$$d(\boldsymbol{\theta}, \boldsymbol{\theta}_0; G, G_0) \stackrel{a.s.}{\to} 0.$$

Let P be the true probability measure and P_I be the emipirical measure of the observed data $D_I = \{(t_i, \boldsymbol{y}_i, \boldsymbol{x}_i) : i = 1, ..., I\}$. Note that $(\boldsymbol{\theta}_0, G_0)$ minimizes the Kullback-Leibler divergence between $\ell(\boldsymbol{\theta}_0, G_0|\boldsymbol{s})$ and $\ell(\boldsymbol{\theta}, G|\boldsymbol{s})$, equivalently it maximizes $P[\ell(\boldsymbol{\theta}, G|\boldsymbol{s})]$, i.e.

$$(\boldsymbol{\theta}_0, G_0) = \arg \max_{(\theta,G)\in(\Theta,\mathcal{G})} P[\ell(\boldsymbol{\theta}, G|\boldsymbol{s})],$$

and since the model is identifiable, $(\boldsymbol{\theta}_0, G_0)$ is the unique maximizer, and $\ell(\boldsymbol{\theta}, G|\boldsymbol{s})$ is continuous in $(\boldsymbol{\theta}, G)$, and $G_0(\cdot)$ is continuous by (C3), thus for any $\delta > 0$,

$$\sup_{d(\theta,\theta_0;G,G_0)>\delta} P[\ell(\boldsymbol{\theta}, G|\boldsymbol{s})] < P[\ell(\boldsymbol{\theta}_0, G_0|\boldsymbol{s})].$$

Also, since $(\hat{\boldsymbol{\theta}}, \hat{G})$ is the MLE,

$$P_I[\ell(\hat{\boldsymbol{\theta}}, \hat{G}|\boldsymbol{s})] \geq P_I[\ell(\boldsymbol{\theta}_0, G_0|\boldsymbol{s})].$$

Thus, by Theorem 5.8 in van der Vaart (2002, p.386), if we can show the class of functions $\mathcal{M} = \{\ell(\boldsymbol{\theta}, G|\boldsymbol{s}) : (\boldsymbol{\theta}, G) \in (\boldsymbol{\Theta}, \mathcal{G})\}$ is P-Glivenko-Cantelli, then $d(\hat{\boldsymbol{\theta}}, \boldsymbol{\theta}_0; \hat{G}, G_0) \stackrel{a.s.}{\to} 0$ and finishing proof.

Now we check $\mathcal{M}$ being a Glivenko-Cantelli class. For $\epsilon > 0$, let $N_{[]}(\epsilon, \mathcal{M}, L_2(P))$ be the minimum number of ϵ-brackets needed to cover $\mathcal{M}$ under the norm $||\cdot||_{L_2(P)}$. By (2),

$$\ell(\boldsymbol{\theta}, G|\boldsymbol{s}) = \sum_{j=1}^{k} y_j\big(\log G(t) + \boldsymbol{\beta}^{\mathsf{T}}\boldsymbol{x}_j\big) - G(t)\sum_{j=1}^{k}\exp(\boldsymbol{\beta}^{\mathsf{T}}\boldsymbol{x}_j)$$

$$+\log\Big(\sum_{|\mathbf{r}|=0}^{J-k}\lambda^{|\mathbf{r}|}(1-\lambda)^{J-k-|\mathbf{r}|}\exp\big\{-G(t)\boldsymbol{r}^{\mathsf{T}}\mathbf{exp}(\boldsymbol{\beta}^{\mathsf{T}}\boldsymbol{x})\big\}\Big).$$

We have, by (C1)-(C6), for $d(\boldsymbol{\theta}_1, \boldsymbol{\theta}_2; G_1, G_2)$ small and some generic $0 < C < \infty$,

$$\big|\ell(\boldsymbol{\theta}_1, G_1|\boldsymbol{s}) - \ell(\boldsymbol{\theta}_2, G_2|\boldsymbol{s})\big| \le C\sum_{j=1}^{k}\Big(\Big|y_j\frac{G_2(t)-G_1(t)}{G_1(t)}\Big| + \big|y_j(\boldsymbol{\beta}_1-\boldsymbol{\beta}_2)^{\mathsf{T}}\boldsymbol{x}_j\big| + \big|G_1(t)-G_2(t)\big|$$

$$+\big|\exp(\boldsymbol{\beta}_1^{\mathsf{T}}\boldsymbol{x}_j) - \exp(\boldsymbol{\beta}_2^{\mathsf{T}}\boldsymbol{x}_j)\big|\Big) + C\sum_{|\mathbf{r}|=0}^{J-k}\Big|\exp\big\{-G_1(t)\boldsymbol{r}^{\mathsf{T}}\mathbf{exp}(\boldsymbol{\beta}_1^{\mathsf{T}}\boldsymbol{x})\big\} - \exp\big\{-G_2(t)\boldsymbol{r}^{\mathsf{T}}\mathbf{exp}(\boldsymbol{\beta}_2^{\mathsf{T}}\boldsymbol{x})\big\}\Big|$$

$$\le C\sum_{j=1}^{k}\Big(\Big|y_j\frac{G_2(t)-G_1(t)}{G_1(t)}\Big| + \big|y_j(\boldsymbol{\beta}_1-\boldsymbol{\beta}_2)^{\mathsf{T}}\boldsymbol{x}_j\big| + \big|G_1(t)-G_2(t)\big| + \big|(\boldsymbol{\beta}_1-\boldsymbol{\beta}_2)^{\mathsf{T}}\boldsymbol{x}_j\big|\Big).$$

Since $E\|\boldsymbol{x}_j\| < \infty$, $E(y_j\|\boldsymbol{x}_j\|) < \infty$, and by (C5), $\sup_{G\in\mathcal{G}_\eta} E([y_j^2/G^2(t)] < \infty$, so

$$\|\ell(\boldsymbol{\theta}_1, G_1|\cdot) - \ell(\boldsymbol{\theta}_2, G_2|\cdot)\|_{L_1(P)} \le C\|G_1 - G_2\|_{L_2(P)} + C\|\boldsymbol{\beta}_1 - \boldsymbol{\beta}_2\|_{L_1(P)},$$

and so

$$N_{[]}(\epsilon, \mathcal{M}, ||\cdot||_{L_1(P)} \le N_{[]}(\frac{\epsilon}{2C}, \mathcal{G}, ||\cdot||_{L_2(P)})N_{[]}(\frac{\epsilon}{2C}, \boldsymbol{B}, ||\cdot||_{L_1(P)}).$$

With (C4) and that $\mathcal{G}$ is a set of bounded and monotone increasing functions on $[0,1]$, by Theorem 2.7.5 in [1],

$$N_{[]}(\frac{\epsilon}{2C_2}, \mathcal{G}, ||\cdot||_{L_2(P)}) \le \exp(\frac{C}{\epsilon}), \quad \forall\epsilon > 0.$$

With (C2) and $\boldsymbol{B}$ is set in Euclidean space, $N_{[]}(\frac{\epsilon}{2C}, \boldsymbol{B}, ||\cdot||_{L_1(P)}) = O(\epsilon^{-d})$, with $d = dim(\boldsymbol{\theta})$, and so

$$N_{[]}(\epsilon, \mathcal{M}, ||\cdot||_{L_1(P)}) \le \frac{C}{\epsilon^d}\exp(\frac{C}{\epsilon}) < \infty, \quad \forall\epsilon > 0.$$

By Theorem 2.4.1 in [1], $\mathcal{M}$ is a Glivenko-Cantelli class, and finishes the proof.

Lemma 1. Let $d(\boldsymbol{\beta}, \boldsymbol{\beta}_0; G, G_0) = ||\boldsymbol{\beta} - \boldsymbol{\beta}_0|| + \sup_{t\in[0,1]} |G(t) - G_0(t)|$, under conditions of Theorem 1,

$$d(\hat{\boldsymbol{\beta}}, \boldsymbol{\beta}_0; \hat{G}, G_0) = O_p(I^{-1/3}).$$

Proof of Lemma 1. In fact we will show $d(\hat{\boldsymbol{\theta}}, \boldsymbol{\theta}_0; \hat{G}, G_0) = O_p(I^{-1/3})$. We willl use Theorem 3.4.1 in van der Vaart and Wellner (1996) to prove the result. Below we check the conditions of this Theorem. Let $\ell(\boldsymbol{\theta}, G|\boldsymbol{s})$ be given in the proof of Theorem 1. Define $M_I(\boldsymbol{\beta}, G) = P_I \ell(\boldsymbol{\theta}, G|\boldsymbol{s})$ and $M(\boldsymbol{\theta}, G) = P\ell(\boldsymbol{\theta}, G|\boldsymbol{s})$.

From Theorem 1, we have $d(\hat{\boldsymbol{\theta}}, \boldsymbol{\theta}_0; \hat{G}, G_0) \xrightarrow{P} 0$. Since $(\hat{\boldsymbol{\theta}}, \hat{G})$ is the MLE, $M_I(\hat{\boldsymbol{\theta}}, \hat{G}) \geq M_I(\boldsymbol{\theta}_0, G_0)$.

Note that $(\boldsymbol{\theta}_0, G_0) = \arg\sup_{(\theta,G)\in(\Theta,\mathcal{G})} M(\boldsymbol{\theta}, G)$. Denote $\dot{\ell}_\theta(\boldsymbol{\theta}_0, G_0|\boldsymbol{s})$, $\dot{\ell}_G(\boldsymbol{\theta}_0, G_0|\boldsymbol{s})[G-G_0]$, $\ddot{\ell}_{\theta,\theta}(\boldsymbol{\theta}, G|\boldsymbol{s})$, $\ddot{\ell}_{\theta,G}(\boldsymbol{\theta}, G|\boldsymbol{s})[G-G_0]$, $\ddot{\ell}_{G,\theta}(\boldsymbol{\theta}, G|\boldsymbol{s})[h]$ and $\ddot{\ell}_{G,G}(\boldsymbol{\theta}, G|\boldsymbol{s})[G-G_0, G-G_0]$ be the partial derivatives of $\ell(\boldsymbol{\theta}, G|\boldsymbol{s})$ with respect to $(\boldsymbol{\theta}, G)$. That for G is in the Hadamard sense. Then $P\{\dot{\ell}_\theta(\boldsymbol{\theta}_0, G_0|\boldsymbol{s})\} = 0$, and $P\{\dot{\ell}_G(\boldsymbol{\theta}_0, G_0)[G-G_0]\} = 0$.

Denote $\ddot{Q}(\boldsymbol{\theta}, G)[G-G_0, G-G_0]$ for the $(d+1)\times(d+1)$ matrix of expected all the second order partial derivatives, then by Taylor expansion,

$$M(\boldsymbol{\theta}, G) - M(\boldsymbol{\theta}_0, G_0) = \frac{1}{2}P\big((\boldsymbol{\theta}-\boldsymbol{\theta}_0, 1)^\intercal \ddot{Q}(\tilde{\boldsymbol{\theta}}, \tilde{G})[G-G_0; g-g_0](\boldsymbol{\theta}-\boldsymbol{\theta}_0, 1)\big),$$

where $(\tilde{\boldsymbol{\theta}}, \tilde{G})$ lies between $(\boldsymbol{\theta}, G)$ and $(\boldsymbol{\theta}_0, G_0)$. It is easy to check that the above is of order $O(d^2(\boldsymbol{\theta}, \boldsymbol{\theta}_0; G, G_0))$.

So for any $0 < \eta_I \to 0$ and any η and $\eta < \infty$ with $\eta_I < \tau \leq \eta$, for some $0 < C < \infty$.

$$\sup_{\tau/2<d(\theta,\theta_0;G,G_0)\leq\tau,(\theta,G)\in(\Theta,\mathcal{G})} M(\boldsymbol{\theta}, G) - M(\boldsymbol{\theta}_0, G_0) \leq -C\tau^2.$$

Next we need to check, for all $0 < \tau_I < \tau \leq \eta$,

$$E sup_{\tau/2<d(\theta,\theta_0;G,G_0)\leq\tau,(\theta,G)\in(\Theta,\mathcal{G})} \sqrt{I}[(M_I - M)(\boldsymbol{\theta}, G) - (M_I - M)(\boldsymbol{\theta}_0, G_0)]^+ \leq C\phi_I(\tau),$$

for some function ϕ_I and some $\alpha < 2$ such that $\phi_I(\tau)/\tau^\alpha$ is decreasing on (τ_I, η). In fact, we check the following slightly stronger condition

$$E sup_{d(\theta,\theta_0;G,G_0)\leq\tau,(\theta,G)\in(\Theta,\mathcal{G})} \sqrt{n}\big|(M_I - M)(\boldsymbol{\theta}, G) - (M_I - M)(\boldsymbol{\theta}_0, G_0)\big| \leq C\phi_I(\tau).$$

Let $q(\boldsymbol{s}|\boldsymbol{\theta}, G) = \ell(\boldsymbol{\theta}, G|\boldsymbol{s}) - \ell(\boldsymbol{\theta}_0, G_0|\boldsymbol{s})$, $\mathbb{G}_I q = \sqrt{I}(P_I - P)q$, $Q = \{q(\boldsymbol{s}|\boldsymbol{\theta}, G) : (\boldsymbol{\theta}, G) \in (\boldsymbol{\Theta}, \mathcal{G}), d(\boldsymbol{\theta}, \boldsymbol{\theta}_0; G, G_0) \leq \tau\}$, and $||\mathbb{G}_I||_Q = \sup_{q\in Q} |\mathbb{G}_I q|$. The above inequality is re-written as

$$E||\mathbb{G}_I||_Q \leq C\phi_I(\tau).$$

Below we check the above condition. Similarly as in the proof of Theorem 1, we can have $N_{[]}(\epsilon, Q, L_2(P)) \leq \exp\{C/\epsilon\}$ for some $0 < C < \infty$, and so

$$J_{[]}(\tau, Q, L_2(P)) := \int_0^\tau \sqrt{1 + \log N_{[]}(\epsilon, Q, L_2(P))}d\epsilon$$

$$\leq \int_0^\tau \sqrt{1 + C/\epsilon}d\epsilon = \int_{-\infty}^{\log\tau} e^s\sqrt{1 + Ce^{-s}}ds \leq C\tau^{1/2}.$$

In the above, we used the fact that for small $\tau > 0$, $\log\tau < 0$, so $e^{-s} > 1$ and $1 + Ce^{-s} \le (1+C)e^{-s}$ on $(-\infty, \log\tau)$.

Since $q(\boldsymbol{s}|\boldsymbol{\theta}, G)$ is continuous in $(\boldsymbol{\theta}, G)$, the condition $d(\boldsymbol{\theta}, \boldsymbol{\theta}_0; G, G_0)$ implies $Pq^2(\boldsymbol{s}|\boldsymbol{\theta}, G) \le c\tau^2$ for all $q \in Q$ for some $0 < c < \infty$; and $\sup_s |q(\boldsymbol{s}|\boldsymbol{\theta}, G)| < M$ for all $q \in Q$ and some $0 < M < \infty$. Now by Lemma 3.4.2 in [1], for some generic $0 < C < \infty$,

$$E||\mathbb{G}_I||_Q \le CJ_{[]}(\tau, Q, L_2(P))\Big(1 + \frac{CJ_{[]}(\tau, Q, L_2(P))}{\tau^2\sqrt{I}}C\Big) \le C\tau^{1/2}(1 + \tau^{-3/2}I^{-1/2}).$$

So wiith $\phi(\tau) = C\tau^{1/2}(1 + \tau^{-3/2}I^{-1/2})$ and $r_I = I^{1/3}$, we have

$$r_I^2\phi_n(\frac{1}{r_I}) = CI^{1/3} \le \sqrt{I}.$$

Now by the Theorem 3.4.1 in [1],

$$I^{1/3}d(\hat{\boldsymbol{\theta}}, \boldsymbol{\theta}_0; \hat{G}, G_0) = O_p(1).$$

Lemma 2. *The efficient score for estimating $\boldsymbol{\theta}_0$ is*

$$\ell_\theta^*(\boldsymbol{\theta}_0, G_0|\boldsymbol{s}) = \dot{\ell}_\theta(\boldsymbol{\theta}_0, G_0|\boldsymbol{s}) - A(\boldsymbol{s}|\boldsymbol{\theta}_0, G_0)\boldsymbol{h}^*(t),$$

$$A(\boldsymbol{s}|\boldsymbol{\theta}_0, G_0) = \sum_{j=1}^{k}\Big[\frac{y_j}{G_0(t)} - \exp(\boldsymbol{\beta}_0^\top \boldsymbol{x}_j)\Big]$$

$$-\frac{\sum_{|\mathrm{r}|=0}^{J-k}\lambda_0^{|\mathrm{r}|}(1-\lambda_0)^{J-k-|\mathrm{r}|}\exp\big\{-G_0(t)\boldsymbol{r}^\top\mathbf{exp}(\boldsymbol{\beta}_0^\top\boldsymbol{x})\big\}\boldsymbol{r}^\top\mathbf{exp}(\boldsymbol{\beta}_0^\top\boldsymbol{x})}{\sum_{|\mathrm{r}|=0}^{J-k}\lambda_0^{|\mathrm{r}|}(1-\lambda_0)^{J-k-|\mathrm{r}|}\exp\big\{-G_0(t)\boldsymbol{r}^\top\mathbf{exp}(\boldsymbol{\beta}_0^\top\boldsymbol{x})\big\}},$$

and

$$\boldsymbol{h}^*(t) = \frac{E\big\{\dot{\ell}_\theta(\boldsymbol{\theta}_0, G_0|\boldsymbol{s})A(\boldsymbol{s}|\boldsymbol{\theta}_0, G_0)\big|T = t\big\}}{E\big\{A^2(\boldsymbol{s}|\boldsymbol{\theta}_0, G_0)\big|T = t\big\}}.$$

Proof of Lemma 2 . *With $\ell(\boldsymbol{\theta}, G|\boldsymbol{s})$ given in the proof of Theorem 1, the Hadamard derivative of $\ell(\boldsymbol{\theta}, G|\boldsymbol{s})$ with respect to $G(\cdot)$ in the direction $h(\cdot)$ is*

$$\dot{\ell}_G(\boldsymbol{\theta}, G|\boldsymbol{s})[h] = A(\boldsymbol{s}|\boldsymbol{\theta}, G)h(t) := \Big(\sum_{j=1}^{k}\Big[\frac{y_j}{G(t)} - \exp(\boldsymbol{\beta}^\top\boldsymbol{x}_j)\Big]$$

$$-\frac{\sum_{|\mathrm{r}|=0}^{J-k}\lambda^{|\mathrm{r}|}(1-\lambda)^{J-k-|\mathrm{r}|}\exp\big\{-G(t)\boldsymbol{r}^\top\mathbf{exp}(\boldsymbol{\beta}^\top\boldsymbol{x})\big\}\boldsymbol{r}^\top\mathbf{exp}(\boldsymbol{\beta}^\top\boldsymbol{x})}{\sum_{|\mathrm{r}|=0}^{J-k}\lambda^{|\mathrm{r}|}(1-\lambda)^{J-k-|\mathrm{r}|}\exp\big\{-G(t)\boldsymbol{r}^\top\mathbf{exp}(\boldsymbol{\beta}^\top\boldsymbol{x})\big\}}\Big)h(t).$$

The efficient score for estimating $\boldsymbol{\theta}_0$ is

$$\ell^*_\theta(\boldsymbol{\theta}_0, G_0|\boldsymbol{s}) = \dot{\ell}_\theta(\boldsymbol{\theta}_0, G_0|\boldsymbol{s}) - \dot{\ell}_G(\boldsymbol{\theta}_0, G_0|\boldsymbol{s})[\boldsymbol{h}^*],$$

where $\boldsymbol{h}^* = (h^*_1, ..., h^*_d)^\intercal$, $d = dim(\boldsymbol{\theta})$, $\dot{\ell}_G(\boldsymbol{\beta}_0, G_0|\boldsymbol{s})[\boldsymbol{h}^*] = (\dot{\ell}_G(\boldsymbol{\theta}_0, G_0|D)[h^*_1], ..., \dot{\ell}_G(\boldsymbol{\theta}_0, G_0|D)[h^*_d])^\intercal$, and the least favorable direction $\boldsymbol{h}^*$ is determined by [2],

$$\forall\, h, \qquad 0 = E\{(\dot{\ell}_\theta(\boldsymbol{\theta}_0, G_0|\boldsymbol{s}) - \dot{\ell}_G(\boldsymbol{\theta}_0, G_0|\boldsymbol{s})[\boldsymbol{h}^*])\dot{\ell}_G(\boldsymbol{\theta}_0, G_0|\boldsymbol{s})[h]\}$$

$$= E\Big(E\Big\{(\dot{\ell}_\theta(\boldsymbol{\theta}_0, G_0|\boldsymbol{s}) - A(\boldsymbol{s}|\boldsymbol{\theta}_0, G_0)\boldsymbol{h}^*(t))A(\boldsymbol{s}|\boldsymbol{\theta}_0, G_0)\Big\}h(t)\Big|t\Big).$$

The above gives

$$E\Big\{(\dot{\ell}_\theta(\boldsymbol{\theta}_0, G_0|\boldsymbol{s}) - A(\boldsymbol{s}|\boldsymbol{\theta}_0, G_0)\boldsymbol{h}^*(t))A(\boldsymbol{s}|\boldsymbol{\theta}_0, G_0)\Big|t\Big\} \equiv 0,$$

or

$$\boldsymbol{h}^*(t) = \frac{E\{\dot{\ell}_\theta(\boldsymbol{\theta}_0, G_0|\boldsymbol{s})A(\boldsymbol{s}|\boldsymbol{\theta}_0, G_0)|t\}}{E\{A^2(\boldsymbol{s}|\boldsymbol{\theta}_0, G_0)|t\}},$$

and

$$\ell^*_\theta(\boldsymbol{\theta}_0, G_0|\boldsymbol{s}) = \dot{\ell}_\theta(\boldsymbol{\theta}_0, G_0|\boldsymbol{s}) - A(\boldsymbol{s}|\boldsymbol{\theta}_0, G_0)\boldsymbol{h}^*(t).$$

Proof of Theorem 2. *We only need to prove the following stronger result*

$$\sqrt{I}(\hat{\boldsymbol{\theta}} - \boldsymbol{\theta}_0) \xrightarrow{D} N(0, I^{*-1}(\boldsymbol{\theta}_0, G_0), \qquad I^*(\boldsymbol{\theta}_0, G_0) = E_{(\theta_0, G_0)}[\ell^*_\theta(\boldsymbol{\theta}_0, G_0|\boldsymbol{s})\ell^{*\intercal}_\theta(\boldsymbol{\theta}_0, G_0|\boldsymbol{s})].$$

Let $\dot{\ell}_G(\boldsymbol{\beta}_0, G_0|\boldsymbol{s})[\boldsymbol{h}^*]$ be given in the proof of Lemma 2. Since $(\boldsymbol{\theta}_0, G_0)$ is the true parameter, we have $P\dot{\ell}_\theta(\boldsymbol{\theta}_0, G_0|\boldsymbol{s}) = 0 = P\dot{\ell}_G(\boldsymbol{\beta}_0, G_0|\boldsymbol{s})[\boldsymbol{h}^*]$, and so $P\dot{\ell}^*_\theta(\boldsymbol{\theta}_0, G_0|\boldsymbol{s}) = P(\dot{\ell}_\theta(\boldsymbol{\theta}_0, G_0|\boldsymbol{s}) - \dot{\ell}_G(\boldsymbol{\theta}_0, G_0|\boldsymbol{s})[\boldsymbol{h}^*]) = 0$. Also since $(\hat{\boldsymbol{\theta}}, \hat{G})$ is the MLE, $P_I\dot{\ell}_\theta(\hat{\boldsymbol{\theta}}, \hat{G}|\boldsymbol{s}) = 0 = P_I\dot{\ell}_G(\hat{\boldsymbol{\theta}}, \hat{G}|\boldsymbol{s})[\boldsymbol{h}^*]$, and so $P_I\dot{\ell}^*_\theta(\hat{\boldsymbol{\theta}}, \hat{G}|\boldsymbol{s}) = P_I(\dot{\ell}_\theta(\hat{\boldsymbol{\theta}}, \hat{G}|\boldsymbol{s}) - \dot{\ell}_G(\hat{\boldsymbol{\theta}}, \hat{G}|\boldsymbol{s})[\boldsymbol{h}^*]) = 0$.

Let $\mathcal{M}_1 = \{\dot{\ell}_\theta(\boldsymbol{\theta}, G|\boldsymbol{s}) : (\boldsymbol{\theta}, G) \in (\boldsymbol{\Theta}, \mathcal{G})\}$ and $\mathcal{M}_2 = \{\dot{\ell}_G(\boldsymbol{\theta}, G|\boldsymbol{s})[\boldsymbol{h}^*] : (\boldsymbol{\theta}, G) \in (\boldsymbol{\Theta}, \mathcal{G})\}$. For some generic constant $0 < C < \infty$, as in the proof of Theorem 1, we have

$$N_{[]}(\epsilon, \mathcal{M}_j, L_2(P)) \le N_{[]}(\frac{\epsilon}{2C_1}, \boldsymbol{B}, L_2(P))N_{[]}(\frac{\epsilon}{2C_2}, \mathcal{G}, L_2(P)), \qquad (j = 1, 2)$$

thus $N_{[]}(\epsilon, \mathcal{M}_j, L_2(P)) \le \exp(C/\epsilon)$, and

$$J_{[]}(1, \mathcal{M}_j, L_2(P)) := \int_0^1 \sqrt{\log N_{[]}(\epsilon, \mathcal{M}_j, L_2(P))}d\epsilon \le \int_0^1 \sqrt{C/\epsilon}d\epsilon < \infty, \qquad (j = 1, 2).$$

So by Theorem 6.8 in [3], $\mathcal{M}_j$ $(j = 1, 2)$ are Donsker classes. Then, by Corollary 2.3.12 in [1],

$$(P_I - P)\dot{\ell}_\theta(\hat{\boldsymbol{\theta}}, \hat{G}|\boldsymbol{s}) - (P_I - P)\dot{\ell}_\theta(\boldsymbol{\theta}_0, G_0|\boldsymbol{s}) = o_p(I^{-1/2}),$$

$$(P_I - P)\dot{\ell}_G(\hat{\boldsymbol{\theta}}, \hat{G}|\boldsymbol{s})[\boldsymbol{h}^*] - (P_I - P)\dot{\ell}_G(\boldsymbol{\theta}_0, G_0|\boldsymbol{s})[\boldsymbol{h}^*] = o_p(I^{-1/2}).$$

Since $P_I\dot{\ell}_\theta(\hat{\boldsymbol{\theta}}, \hat{G}|\boldsymbol{s}) = 0$ and $P\dot{\ell}_\theta(\boldsymbol{\theta}_0, G_0|\boldsymbol{s}) = 0$, the above first equation gives

$$P\dot{\ell}_\theta(\hat{\boldsymbol{\theta}}, \hat{G}|\boldsymbol{s}) = -P_I\dot{\ell}_\theta(\boldsymbol{\theta}_0, G_0|\boldsymbol{s}) + o_p(I^{-1/2}). \qquad (A.1)$$

Similarly, since $P_I\dot{\ell}_G(\hat{\boldsymbol{\theta}}_0, \hat{G}|\boldsymbol{s})[\boldsymbol{h}^*] = 0$ and $P\dot{\ell}_G(\boldsymbol{\theta}_0, G_0|\boldsymbol{s})[\boldsymbol{h}^*] = 0$. The above second equation gives

$$P\dot{\ell}_G(\hat{\boldsymbol{\theta}}, \hat{G}|\boldsymbol{s})[\boldsymbol{h}^*] = -P_I\dot{\ell}_G(\boldsymbol{\theta}_0, G_0|\boldsymbol{s})[\boldsymbol{h}^*] + o_p(I^{-1/2}). \qquad (A.2)$$

Also, by Lemma 1, $E(||\hat{\theta} - \boldsymbol{\theta}_0||^2 + ||\hat{G} - G_0||^2) = O(I^{-2/3})$, so by Taylor expansion,

$$P\{\dot{\ell}_\theta(\hat{\boldsymbol{\theta}}, \hat{G}|\boldsymbol{s}) - \dot{\ell}_\theta(\boldsymbol{\theta}_0, G_0|\boldsymbol{s}) - \ddot{\ell}_{\theta\theta}(\boldsymbol{\theta}_0, G_0|\boldsymbol{s})(\hat{\boldsymbol{\theta}} - \boldsymbol{\theta}_0) - \ddot{\ell}_{\theta,G}(\boldsymbol{\theta}_0, G_0)[\hat{G} - G_0]\} = O(I^{-2/3}).$$

Since $P\dot{\ell}_\theta(\boldsymbol{\theta}_0, G_0|\boldsymbol{s}) = 0$, we get

$$P\{\dot{\ell}_\theta(\hat{\boldsymbol{\theta}}, \hat{G}|\boldsymbol{s}) - \ddot{\ell}_{\theta\theta}(\boldsymbol{\theta}_0, G_0|\boldsymbol{s})(\hat{\boldsymbol{\theta}} - \boldsymbol{\theta}_0) - \ddot{\ell}_{\theta,G}(\boldsymbol{\theta}_0, G_0|\boldsymbol{s})[\hat{G} - G_0]\} = o_p(I^{-1/2}), \qquad (A.3)$$

Similarly,

$$P\{\dot{\ell}_G(\boldsymbol{\theta}_0, G_0|\boldsymbol{s})[\boldsymbol{h}^*] - \ddot{\ell}_{G,\theta}(\boldsymbol{\theta}_0, G_0|\boldsymbol{s})[\boldsymbol{h}^*](\hat{\boldsymbol{\theta}} - \boldsymbol{\theta}_0) - \ddot{\ell}_{G,G}(\boldsymbol{\theta}_0, G_0|\boldsymbol{s})[\boldsymbol{h}^*, \hat{G} - G_0]\} = o_p(I^{-1/2}). \quad (A.4)$$

Also, by definition of $\boldsymbol{h}^*$,

$$P\ddot{\ell}_{\theta,G}(\boldsymbol{\theta}_0, G_0|\boldsymbol{s})[\hat{G} - G_0] - P\ddot{\ell}_{G,G}(\boldsymbol{\theta}_0, G_0|\boldsymbol{s})[\boldsymbol{h}^*, \hat{G} - G_0]$$

$$= -P\big(\dot{\ell}_\theta(\boldsymbol{\theta}_0, G_0|\boldsymbol{s})\dot{\ell}_G(\boldsymbol{\theta}_0, G_0|\boldsymbol{s})[\hat{G} - G_0]\big) - P\big(\dot{\ell}_G(\boldsymbol{\theta}_0, G_0|\boldsymbol{s})[\boldsymbol{h}^*]\dot{\ell}_G(\boldsymbol{\theta}_0, G_0|\boldsymbol{s})[\hat{G} - G_0]\big)$$

$$= -P\big((\dot{\ell}_\theta(\boldsymbol{\theta}_0, G_0|\boldsymbol{s}) - \dot{\ell}_G(\boldsymbol{\theta}_0, G_0|\boldsymbol{s})[\boldsymbol{h}^*])\dot{\ell}_G(\boldsymbol{\theta}_0, G_0|\boldsymbol{s})[\hat{G} - G_0]\big) = 0.$$

Now (A.1)-(A.4) gives

$$P(\ddot{\ell}_{\theta\theta}(\boldsymbol{\theta}_0, G_0|\boldsymbol{s}) - \ddot{\ell}_{\theta,G}(\boldsymbol{\theta}_0, G_0|\boldsymbol{s})[\boldsymbol{h}^*])(\hat{\boldsymbol{\theta}} - \boldsymbol{\theta}_0) = -P_I(\dot{\ell}_\theta(\boldsymbol{\theta}_0, G_0|\boldsymbol{s}) - \dot{\ell}_G(\boldsymbol{\theta}_0, G_0|\boldsymbol{s})[\boldsymbol{h}^*]) + o_p(I^{-1/2}). \qquad (A.5)$$

Since

$$P(\ddot{\ell}_{\theta\theta}(\boldsymbol{\theta}_0, G_0|\boldsymbol{s}) - \ddot{\ell}_{\theta,G}(\boldsymbol{\theta}_0, G_0|\boldsymbol{s})[\boldsymbol{h}^*]) = -P\big((\dot{\ell}_\theta(\boldsymbol{\theta}_0, G_0|\boldsymbol{s})(\dot{\ell}_\theta(\boldsymbol{\theta}_0, G_0|\boldsymbol{s}) - \dot{\ell}_G(\boldsymbol{\theta}_0, G_0|\boldsymbol{s})[\boldsymbol{h}^*])^\top\big)$$

$$= -P\big((\dot{\ell}_\theta(\boldsymbol{\theta}_0, G_0|\boldsymbol{s}) - \dot{\ell}_G(\boldsymbol{\theta}_0, G_0|\boldsymbol{s})[\boldsymbol{h}^*])(\dot{\ell}_\theta(\boldsymbol{\theta}_0, G_0|\boldsymbol{s}) - \dot{\ell}_G(\boldsymbol{\theta}_0, G_0|\boldsymbol{s})[\boldsymbol{h}^*])^\top\big)$$

$$= P(\dot{\ell}^*_\theta(\boldsymbol{\theta}_0, G_0|\boldsymbol{s})(\dot{\ell}^*_\theta(\boldsymbol{\theta}_0, G_0|\boldsymbol{s})^\top) := -I^*(\boldsymbol{\theta}_0, G_0),$$

and $\dot{\ell}_\theta(\boldsymbol{\theta}_0, G_0|\boldsymbol{s}) - \dot{\ell}_G(\boldsymbol{\theta}_0, G_0|\boldsymbol{s})[\boldsymbol{h}^*] = \dot{\ell}^*_\theta(\boldsymbol{\theta}_0, G_0|\boldsymbol{s})$*, (A.5) becomes*

$$\sqrt{I}(\hat{\boldsymbol{\theta}} - \boldsymbol{\theta}_0) = I^{*-1}(\boldsymbol{\theta}_0, G_0)\sqrt{I}P_I\dot{\ell}^*_\theta(\boldsymbol{\theta}_0, G_0|\boldsymbol{s}) + o_p(1).$$

This gives the desired result.

Proof of Theorem 3. *By the algorithm given in (5) of Section 3, with* $r \to \infty$*, we have*

$$\hat{G} = \arg\min_{G\in\mathcal{G}} \frac{1}{I}\sum_{i=1}^{I} \hat{d}_i\big(\frac{y_{i\cdot}}{\hat{d}_i} - G(t_i)\big)^2,$$

where $\hat{d}_i = \sum_{j=1}^{k_i}\exp(\hat{\boldsymbol{\beta}}^\top\boldsymbol{x}_i) + \sum_{j=k_i+1}^{J}\hat{\delta}_{ij}\exp(\hat{\boldsymbol{\beta}}^\top\boldsymbol{x}_{ij})$*. Let* $d_i = \sum_{j=1}^{k_i}\exp(\boldsymbol{\beta}_0^\top\boldsymbol{x}_i) + \sum_{j=k_i+1}^{J}\hat{\delta}_{ij}\exp(\boldsymbol{\beta}_0^\top\boldsymbol{x}_{ij})$*, here* $\hat{\delta}_{ij}$ *depends on* I*, and as* $I \to \infty$ *we still denote the limit as* $\hat{\delta}_{ij}$ *without confusion. By Theorem 2,* $\|\hat{\boldsymbol{\beta}} - \boldsymbol{\beta}_0\| = O_p(I^{-1/2})$*, so we have* $\hat{d}_i = d_i + O(I^{-1/2})$ *uniformly in* i*, and so*

$$\hat{G} = \arg\min_{G\in\mathcal{G}} \frac{1}{I}\sum_{i=1}^{I} d_i\big(\frac{y_{i\cdot}}{d_i} - G(t_i)\big)^2 + O_p(I^{-1/2}) := \tilde{G} + O_p(I^{-1/2}).$$

Since $\|\hat{G} - \tilde{G}\| = I^{-1/2}$*,* $I^{1/3}(\hat{G}(t) - G_0(t))$ *and* $I^{1/3}(\tilde{G}(t) - G_0(t))$ *have the same asymptotic distribution, in this sense we can just write*

$$\hat{G} = \arg\min_{G\in\mathcal{G}} \frac{1}{I}\sum_{i=1}^{I} d_i\big(\frac{y_{i\cdot}}{d_i} - G(t_i)\big)^2$$

$$= \arg\min_{G\in\mathcal{G}} \frac{1}{I}\sum_{i=1}^{I} d_i\big(G(t_i) - G_0(t_i) + G_0(t_i) - \frac{y_{i\cdot}}{d_i}\big)^2$$

$$= \arg\min_{G\in\mathcal{G}} \frac{1}{I}\sum_{i=1}^{I} d_i\big(G(t_i) - G_0(t_i) - \epsilon_i\big)^2,$$

where $\epsilon_i = y_{i\cdot}/d_i - G_0(t_i)$*. Note that* $E(\epsilon_i) = E\{E(\epsilon_i)|t_i, \boldsymbol{x}_i, \hat{\boldsymbol{\delta}}_i\} = E\{E[y_{i\cdot}/d_i - G_0(t_i)]|t_i, \boldsymbol{x}_i, \hat{\boldsymbol{\delta}}_i\} = \sum_{j=1}^{J} E\{E[y_{ij}/d_i - G_0(t_i)]|t_i, \boldsymbol{x}_{ij}, \hat{\boldsymbol{\delta}}_i\} = E\{G_0(t_i)d_i/d_i - G_0(t_i)\} = 0.$

As is typical, let upper letters stands for random variables and lower letters for their observations. Define

$$U_I(t) = P_I[DI(T \le t)] = \frac{1}{I}\sum_{i=1}^{I} d_i I(t_i \le t),$$

$$V_I(t) = P_I[D(G_0(T)+\epsilon)I(T \le t)] = \frac{1}{I}\sum_{i=1}^{I} d_i(G_0(t_i)+\epsilon_i)I(t_i \le t).$$

By Example 3.2.15 in [1], for all $t, a \in R$, $\hat{G}(t) \le a$ *iff* $\arg\min_s\{V_I(s) - aU_I(s)\} \ge t$.

To derive the asymptotic distribution of $I^{1/3}(\hat{G}(t) - G_0(t))$, *we only need to compute the limit of the probability* $P\big(I^{1/3}(\hat{G}(t) - G_0(t)) \le c\big)$ *for arbitary* c. *Note that* $\{I^{1/3}(\hat{G}(t) - G_0(t)) \le c\} = \{\hat{G}(t) \le G_0(t) + I^{-1/3}c\}$. *So, with* $a = G_0(t) + cI^{-1/3}$,

$$P\Big(\hat{G}(t) \le G_0(t) + cI^{-1/3}\Big) = P\Big(\arg\min_s\{V_I(s) - (G_0(t)+cI^{-1/3})U_I(s)\} - t \ge 0\Big).$$

By the change of variable $s = t + I^{-1/3}h$, *we get*

$$\arg\min_s \big\{V_I(s) - (G_0(t)+cI^{-1/3})U_I(s)\big\} - t$$

$$= I^{-1/3}\arg\min_h \big\{V_I(t+I^{-1/3}h) - (G_0(t)+cI^{-1/3})U_I(t+I^{-1/3}h)\big\}.$$

Let $\hat{h}_I$ *be the above minimizer. From above,* $P\big(I^{1/3}(\hat{G}(t) - G_0(t)) \le c\big) = P(\hat{h}_I \ge 0)$. *Note that*

$$\hat{h}_I = \arg\min_h\{V_I(t+I^{-1/3}h) - (G_0(t)+cI^{-1/3})U_I(t+I^{-1/3}h)\}$$

$$= \arg\min_h\{P_I[D(G_0(T)+\epsilon)I(T \le t+I^{-1/3}h)]$$

$$-(G_0(t)+cI^{-1/3})P_I[DI(T \le t+I^{-1/3}h)]\}$$

$$= \arg\min_h\{P_I[D(G_0(T)-G_0(t)+\epsilon)I(T \le t+I^{-1/3}h)]$$

$$-cI^{-1/3}P_I[DI(T \le t+I^{-1/3}h)]\}$$

$$= \arg\min_h\{P_I[D(G_0(T)-G_0(t)+\epsilon)I(t < T \le t+I^{-1/3}h)]$$

$$-cI^{-1/3}P_I[DI(t < T \le t + I^{-1/3}h)]\}$$

$$= \arg\min_h\{(P_I - P)[D(G_0(T) - G_0(t) + \epsilon)I(t < C \le t + I^{-1/3}h)]$$

$$-cI^{-1/3}P_I[DI(t < T \le t + I^{-1/3}h)]$$

$$+P[D(G_0(T) - G_0(t) + \epsilon)I(t < T \le t + I^{-1/3}h)]\}$$

$$= \arg\min_h\{I^{2/3}(P_I - P)[D(G_0(T) - G_0(t) + \epsilon)I(t < T \le t + I^{-1/3}h)]$$

$$-cI^{1/3}P_I[DI(t < T \le t + I^{-1/3}h)]$$

$$+I^{2/3}P[D(G_0(T) - G_0(t) + \epsilon)I(t < T \le t + I^{-1/3}h)]\}$$

$$:= \arg\min_h\{B_{1,I}(h) + B_{2,I}(h) + B_{3,I}(h)\}.$$

Below we evaluate the $B_{j,I}(h)$*'s. We have*

$$B_{1,I}(h) = I^{2/3}(P_I - P)[D(G_0(T) - G_0(t) + \epsilon)I(t < T \le t + I^{-1/3}h)]$$

$$= I^{1/2}I^{1/6}(P_I - P)[D(G_0(T) - G_0(t) + \epsilon)I(t < T \le t + I^{-1/3}h)] = I^{1/2}(P_I - P)r_{I,h}(S),$$

where, $r_{I,h}(S) = I^{1/6}[D(G_0(T) - G_0(t) + \epsilon)I(t < T \le t + I^{-1/3}h)]$.

Let $\mathcal{R} = \{r_{I,h} : h \in [-K, K]\}$ *for some* $0 < K < \infty$*. By (C1), (C2) and (C4),* D *and* $G_0(\cdot)$ *are bounded, so* $\mathcal{R}$ *has an envelop* $r_I(S) = I^{1/6}(C + |\epsilon|)I[t - KI^{-1/3} < T \le t + KI^{-1/3}]$ *for some* $0 < C < \infty$*. Below we check the conditions of Theorem 2.11.23 in [1]. Note that the* y_{ij}*'s are Poisson counts, so* $y_{i\cdot}$ *has finite second moment, which imply that of* ϵ_i*. Thus for some generic* $0 < C < \infty$,

$$\begin{aligned} Pr_I^2 &= I^{1/3}E(C + |\epsilon|)I[t - KI^{-1/3} < T \le t + KI^{-1/3}]) \\ &\le I^{1/3}E(I[t - KI^{-1/3} < T \le t + KI^{-1/3}]) \\ &= I^{1/3}C\int_{t-KI^{-1/3}}^{t+KI^{-1/3}} f(T)d \sim 2CKf(t), \end{aligned}$$

so $Pr_I^2 = O(1)$.

Since $r_I(S)$ has finite second moment, and for each $\eta > 0$, $I(r_I(S) > \eta\sqrt{I}) \to 0$, we have $Pr_I^2 I(r_I > \eta\sqrt{I}) \to 0$. Also,

$$Pr_{I,h}(S) = I^{1/6}E[E[D|T=t](G_0(T) - G_0(t) + \epsilon)I(t < T \le t + I^{-1/3}h)] = O(I^{-1/6}) \to 0,$$

and with $D_2(t) := E[D^2|T=t]$, and $\eta^2(t) = E(\epsilon^2|T=t)$,

$$P[r_{I,h_1}(S)r_{I,h_2}(S)] = I^{1/3}E\{D^2(G_0(T) - G_0(t) + \epsilon)^2 I(t < T \le t + I^{-1/3}(h_1 \wedge h_2)\}$$

$$= I^{1/3}E\{E(D^2(G_0(T) - G_0(t) + \epsilon)^2 I(t < T \le t + I^{-1/3}(h_1 \wedge h_2)|T)\}$$

$$= I^{1/3}E\{E(D^2[(G_0(T) - G_0(t))^2 + 2(G_0(T) - G_0(t))\epsilon + \epsilon^2]I(t < T \le t + I^{-1/3}(h_1 \wedge h_2)|T)\}$$

$$\sim I^{1/3}E\{E(D^2[\dot{G}_0^2(t)(T-t)^2 + 2\dot{G}_0(t)(T-t)\epsilon + \epsilon^2]I(t < T \le t + I^{-1/3}(h_1 \wedge h_2)|T)\}$$

$$\sim I^{1/3}E\{E(D^2\epsilon^2 I(t < T \le t + I^{-1/3}(h_1 \wedge h_2)|T)\}$$

$$= I^{1/3}\int_t^{t+I^{-1/3}(h_1 \wedge h_2)} E(D^2\epsilon^2|T=s)f(s)ds.$$

It is typical to assume that conditioning on T, D and ϵ are independent, so with condition (C9) the above is

$$= I^{1/3}\int_t^{t+I^{-1/3}(h_1 \wedge h_2)} D_2(s)\epsilon^2(s)f(s)ds \sim (h_1 \wedge h_2)D_2(t)f(t)\eta^2(t).$$

Thus as $I \to \infty$, $P[r_{I,h_1}(t)r_{I,h_2}(t)] \sim (h_1 \wedge h_2)D_2(t)f(t)\eta^2(t)$. This gives $Cov(r_{I,h_1}, r_{I,h_2})$ converges to the covariance function of the process $\eta(t)D_2^{1/2}(t)f^{1/2}(t)\mathbb{B}(\cdot)$, where $\mathbb{B}(h)$ is a mean zero Gaussian process on R with covariance function $E[\mathbb{B}(h_1)\mathbb{B}(h_2)] = h_1 \wedge h_2$.

Also, similarly as in the proof of Lemma 1, it can be checked that $\int_0^{\delta_n} \sqrt{N_{[]}(\epsilon||\mathcal{R}||_{P,2}, \mathcal{R}, L_2(P))}d\epsilon \to 0$ for every $\delta_n \to 0$, thus by Theorem 2.11.23 in [1],

$$B_{1,I}(h) \xrightarrow{D} \eta(t)D_2^{1/2}(t)f^{1/2}(t)\mathbb{B}(h), \quad in \ \ell^\infty[-K, K].$$

It can be shown, as in [1], $\hat{h} = \arg\min_h\{B_{1,I}(h) + B_{2,I}(h) + B_{3,I}(h)\}$ is bounded in probability, and so the above weak convergence can be regarded as in $\ell^\infty(R)$.

Now we handle $B_{2,I}(h)$ and $B_{3,I}(h)$. With $D_1(t) = E(D|T = t]$,

$$\begin{aligned}
B_{2,I}(h) &= -cI^{1/3}P_I[DI(t < T \le t + I^{-1/3}h)] \\
&= -cI^{1/3}(D_1(t) + o(1)) \int_t^{t+I^{-1/3}h} f(s)ds \to -cD_1(t)f(t)h; \\
B_{3,I}(h) &= I^{2/3}P[D_1(T)(G_0(T) - G_0(t) + \epsilon)I(t < T \le t + I^{-1/3}h)] \\
&= I^{2/3}(D_1(t) + o(1)) \int_t^{t+I^{-1/3}h} (G_0(s) - G_0(t))f(s)ds \\
&= I^{2/3}(D_1(t) + o(1))(f(t) + o(1)) \int_t^{t+I^{-1/3}h} \dot{G}_0(t)(s-t)ds \\
&\to \frac{1}{2}D_1(t)f(t)\dot{G}_0(t)h^2.
\end{aligned}$$

Now combine the asymptotic results of $B_{1,I}(h)$, $B_{2,I}(h)$ and $B_{3,I}(h)$, we have

$$\begin{aligned}
&\arg\min_h\{B_{1,I}(h) + B_{2,I}(h) + B_{3,I}(h)\} \\
&\overset{D}{\to} \arg\min_h\{\eta(t)D_2^{1/2}(t)f^{1/2}(t)\mathbb{B}(h) - cD_1(t)f(t)h + \frac{1}{2}D_1(t)f(t)\dot{G}_0(t)h^2\}.
\end{aligned}$$

Using Problem 3.2.5 in [1], the above is re-written as

$$\left(\frac{4\eta^2(t)D_2(t)f(t)}{[D_1(t)f(t)\dot{G}_0(t)]^2}\right)^{1/3} \arg\min_h\{\mathbb{B}(h) - h^2\} + \frac{c}{\dot{G}_0(t)}.$$

Finally we have, for all c,

$$\begin{aligned}
&P\big(I^{1/3}(\hat{G}(t) - G_0(t)) \le c\big) \\
&= P\Big(\left(\frac{4\eta^2(t)D_2(t)f(t)}{[D_1(t)f(t)\dot{G}_0(t)]^2}\right)^{1/3} \arg\min_h\{\mathbb{B}(h) - h^2\} + \frac{c}{\dot{G}_0(t)} \ge 0\Big) \\
&= P\Big(\left(\frac{4\eta^2(t)D_2(t)f(t)\dot{G}_0(t)}{[D_1(t)f(t)]^2}\right)^{1/3} \arg\max_h\{-\mathbb{B}(h) + h^2\} \le c\Big) \\
&= P\Big(\left(\frac{4\eta^2(t)D_2(t)f(t)\dot{G}_0(t)}{(D_1(t)f(t))^2}\right)^{1/3} \arg\min_h\{\mathbb{B}(h) + h^2\} \le c\Big) \\
&= P\Big(\left(\frac{4\eta^2(t)D_2(t)\dot{G}_0(t)}{D_1^2(t)f(t)}\right)^{1/3} \arg\min_h\{\mathbb{B}(h) + h^2\} \le c\Big),
\end{aligned}$$

which completes the proof.

Proof of Theorem 4 *As in the proof of Theorem 3, and using notations given in there, for $c \in R$,*

$$\{I^{k/(2k+1)}(\hat{G}_0(t) - G_0(t)) \le c\} = \{\hat{G}_0(t) \le G_0(t) + I^{-k/(2k+1)}c\}.$$

So, with $a = G_0(t) + cI^{-k/(2k+1)}$,

$$P\big(\hat{G}(t) \le G_0(t) + cI^{-k/(2k+1)}\big) = P\big(\arg\min_s\{V_I(s) - (G_0(t) + cI^{-k/(2k+1)})U_I(s)\} - t \ge 0\big)$$

By the change of variable $s = t + I^{-1/(2k+1)}h$, we get

$$\arg\min_s \{V_I(s) - (G_0(t) + cI^{-k/(2k+1)})U_I(s)\} - t$$

$$= I^{-1/(2k+1)} \arg\min_h \{V_I(t + I^{-1/(2k+1)}h) - (G_0(t) + cI^{-k/(2k+1)})U_I(t + I^{-1/(2k+1)}h)\}$$

Let $\hat{h}_I$ be the above minimizer. From above, we need to compute the limit of $P(\hat{h}_I \geq 0)$. Similarly as in the proof of Theorem 3, we have

$$\hat{h}_I = \arg\min_h \{V_I(t + I^{-1/(2k+1)}h) - (G_0(t) + cI^{-k/(2k+1)})U_I(t + I^{-1/(2k+1)}h)\}$$

$$= \arg\min_h \{P_I[D(T_i)(G_0(T_i) + \epsilon)I(T \leq t + I^{-1/(2k+1)}h)]$$

$$-(G_0(t) + cI^{-k/(2k+1)})P_I[DI(T \leq t + I^{-1/(2k+1)}h)]\}$$

$$= \arg\min_h \{P_I[D(G_0(T) - G_0(t) + \epsilon)I(T \leq t + I^{-1/(2k+1)}h)]$$

$$-cI^{-k/(2k+1)}P_I[DI(T \leq t + I^{-1/(2k+1)}h)]\}$$

$$= \arg\min_h \{P_I[D(G_0(T) - G_0(t) + \epsilon)I(t < T \leq t + I^{-1/(2k+1)}h)]$$

$$-cI^{-k/(2k+1)}P_I[DI(t < T \leq t + I^{-1/(2k+1)}h)]\}$$

$$= \arg\min_h \{(P_I - P)[D(G_0(T) - G_0(t) + \epsilon)I(t < C \leq t + I^{-1/(2k+1)}h)]$$

$$-cI^{-k/(2k+1)}P_I[DI(t < T \leq t + I^{-1/(2k+1)}h)]$$

$$+P[w(T)(G_0(T) - G_0(t) + \epsilon)I(t < T \leq t + I^{-1/(2k+1)}h)]\}$$

$$= \arg\min_h \{I^{(k+1)/(2k+1)}(P_I - P)[D(T)(G_0(T) - G_0(t) + \epsilon)I(t < T \leq t + I^{-1/(2k+1)}h)]$$

$$-cI^{1/(2k+1)}P_I[DI(t < T \le t + I^{-1/(2k+1)}h)]$$

$$+I^{(k+1)/(2k+1)}P[D(G_0(T) - G_0(t) + \epsilon)I(t < T \le t + I^{-1/(2k+1)}h)]\}$$

$$:= \arg\min_h \{B_{1,I}(h) + B_{2,I}(h) + B_{3,I}(h)\}.$$

We have

$$\begin{aligned}
B_{3,I}(h) &= I^{(k+1)/(2k+1)}P[D(G_0(T) - G_0(t) + \epsilon)I(t < T \le t + I^{-1/(2k+1)}h)] \\
&= I^{(k+1)/(2k+1)}(D_1(t) + o(1)) \int_t^{t+I^{-1/(2k+1)}h} (G_0(s) - G_0(t))f(s)ds \\
&= I^{(k+1)/(2k+1)}(D_1(t) + o(1))(f(t) + o(1)) \int_t^{t+I^{-1/(2k+1)}h} G^{(k)}(t)(s-t)^k/k!ds \\
&\sim D_1(t)f(t)G^{(k)}(t)h^{k+1}/(k+1)!; \\
B_{2,I}(h) &= -cI^{1/(2k+1)}P_I[DI(t < T \le t + I^{-1/(2k+1)}h)] \\
&\sim -cI^{1/(2k+1)}(D_1(t) + o(1)) \int_t^{t+I^{-1/(2k+1)}h} f(s)ds \sim -cD_1(t)f(t)h;
\end{aligned}$$

$$B_{1,I}(h) = I^{(k+1)/(2k+1)}(P_I - P)[D(G_0(T) - G_0(t) + \epsilon)I(t < T \le t + I^{-1/(2k+1)}h)].$$

Define

$$r_{I,h}(S) = I^{1/2(2k+1)}D(G_0(T) - G_0(t) + \epsilon)I(t < x \le t + I^{-1/(2k+1)}h),$$

then $B_{1,I}(h) = I^{1/2}(P_I - P)r_{I,h}(S)$.

Let $\mathcal{R} = \{r_{I,h} : h \in [-K, K]\}$ *for some* $0 < K < \infty$. *Then* $\mathcal{R}$ *has an envelop* $r_I(S) = I^{1/2(2k+1)}(C + |\epsilon|)I[t - KI^{-1/(2k+1)} < T \le t + KI^{-1/(2k+1)}]$ *with some* $0 < C < \infty$. *Also, similarly as in the proof of Theorem 3,*

$$P[r_{I,q}(S)r_{I,r}(S)] \sim I^{1/(2k+1)}E\int_t^{t+(r\wedge q)I^{-1/(2k+1)}} D^2(G_0(s) - G_0(t) + \epsilon)^2 f(s)ds$$

$$\sim D_2(t)\eta^2(t)f(t)(r \wedge q),$$

and $B_{1,n}(h) \xrightarrow{D} D_2^{1/2}(t)\eta(t)f^{1/2}(t)\mathbb{B}(h)$.

Thus as before,

$$\begin{aligned}
&\arg\min_h \{B_{1,I}(h) + B_{2,I}(h) + B_{3,I}(h)\} \\
&\xrightarrow{D} \arg\min_h \{\eta(t)D_2^{1/2}(t)f^{1/2}(t))\mathbb{B}(h) - cD_1(t)f(t)h + D_1(t)f(t)G^{(k)}(t)h^{k+1}/(k+1)!\}.
\end{aligned}$$

From the above we get, for all $c \in R$,

$$P\big(I^{k/(2k+1)}(\hat{G}_0(t) - G_0(t)) \le c\big)$$

$$\to P\Big(\arg\min_h \{\eta(t) D_2^{1/2}(t) f(t)^{1/2} \mathbb{B}(h) - c D_1(t) f(t) h + D_1(t) f(t) G^{(k)}(t) h^{k+1}/(k+1)!\} \ge 0\Big),$$

and completes the proof.

Web Appendix B

Simulation results: Estimation of parameters

Table 1 shows that bias and standard deviation of the proposed model under Case 1-3 when sample size, dimension of covariates $\boldsymbol{x}$ *and shape of* $G(t)$ *varies. When the sample size is* 200×10*, bias and standard deviation increase as the dimension of covariates increases. Bias and the standard deviation are stable as the shape of* $G(t)$ *varies. Similar conclusion holds for other cases* (1000×100 *and* 5000×10)*. In addition, the bias of the estimates and their standard deviations (sd) decrease as the sample size increases from* 200×10 *to* 1000×100*. In general, most of the results are stable except for some extreme cases with a significant bias up to 0.048 and standard deviation up to 0.128.*

Figures 1, 2 and 3 present the estimates of $G(\cdot)$ *within different cases. Solid blue lines are true shapes of* $G(\cdot)$*, and black step lines are estimates of* $G(\cdot)$ *. Figures show that the lines represent the estimates of* $G(\cdot)$ *and* $G(\cdot)$ *almost overlap, which means the estimates are accurate and stable for different patterns of background noise* $G(\cdot)$*.*

In general, the simulation results show that the estimates of parameters and non-parametric components with the proposed approach are accurate and precise under various background noise patterns and covariates types, which later form the basis of the signal detection process.

TABLE 1 Parameter estimation of the proposed model under Case 1-3 when sample size, dimension of $\boldsymbol{x}$ or shape of $G(t)$ varies.

	Shapes of $G(\cdot)$			
	Convex	*Concave*	$I_{t<0.5}$*Convex*+$I_{t\geq0.5}$*Concave*	$I_{t<0.2}$*Concave*+$I_{t\geq0.2}$*Convex*
	Case 1: $(I = 200; J = 10)$			
$\boldsymbol{\beta}_0$[i]	(2.300,3.500,1.800)	(1.200,2.900,0.600)	(2.400,2.300,1.600)	(1.700,2.100,1.200)
$\hat{\boldsymbol{\beta}}$[ii]	(2.301,3.505,1.793)	(1.200,2.900,0.600)	(2.403,2.306,1.596)	(1.701,2.102,1.198)
sd[iii]	[0.010,0.013,0.013]	[0.002,0.002,0.003]	[0.003,0.003,0.003]	[0.005,0.006,0.004]
$\boldsymbol{\beta}_0$	(-0.600,3.300,2.800)	(-1.100,2.900,1.800)	(-2.400,3.300,2.100)	(-0.900,2.300,3.400)
$\hat{\boldsymbol{\beta}}$	(-0.585,3.305,2.772)	(-1.099,2.902,1.799)	(-2.363,3.326,2.075)	(-0.895,2.304,3.386)
sd	[0.019,0.029,0.031]	[0.004,0.005,0.005]	[0.040,0.035,0.039]	[0.009,0.013,0.013]
$\boldsymbol{\beta}_0$	(-1.300,2.300,2.600)	(2.200,-2.300,3.600)	(2.500,-0.300,3.200)	(3.600,-0.400,4.100)
$\hat{\boldsymbol{\beta}}$	(-1.265,2.307,2.546)	(2.174,-2.141,3.441)	(2.496,-0.280,3.159)	(3.577,-0.389,4.058)
sd	[0.034,0.029,0.043]	[0.123,0.177,0.226]	[0.036,0.039,0.070]	[0.051,0.053,0.077]
$\boldsymbol{\beta}_0$	(1.400,5.300,-2.600)	(1.200,3.700,-1.800)	(2.600,1.500,-2.100)	(1.700,4.300,-2.700)
$\hat{\boldsymbol{\beta}}$	(1.404,5.308,-2.601)	(1.200,3.700,-1.799)	(2.598,1.509,-2.097)	(1.701,4.302,-2.698)
sd	[0.011,0.021,0.012]	[0.002,0.003,0.003]	[0.010,0.009,0.010]	[0.004,0.006,0.006]
$\boldsymbol{\beta}_0$	(2.300,-0.600,2.400,3.100)	(-1.200,2.900,0.700,1.100)	(-0.300,1.200,2.100,2.100)	(2.800,-1.300,2.700,1.400)
$\hat{\boldsymbol{\beta}}$	(2.284,-0.601,2.383,3.097)	(-1.201,2.900,0.700.1.100)	(-0.308,1.207,2.098,2.104)	(2.821,-1.323,2.696,1.399)
sd	[0.042,0.023,0.014,0.025]	[0.008,0.004,0.004,0.002]	[0.013,0.008,0.005,0.007]	[0.025,0.016,0.015,0.010]
$\boldsymbol{\beta}_0$	(-3.900,3.600,-1.300,2.100)	(3.200,2.900,3.100,-1.100)	(3.300,-1.400,3.700,2.800)	(-4.100,3.300,2.600,2.400)
$\hat{\boldsymbol{\beta}}$	(-3.913,3.610,-1.290,2.091)	(3.166,2.923,3.104,-1.096)	(3.301,-1.405,3.699,2.812)	(-4.101,3.301,2.600,2.401)
sd	[0.034,0.027,0.014,0.019]	[0.054,0.031,0.039,0.008]	[0.032,0.018,0.012,0.014]	[0.005,0.003,0.003,0.004]
$\boldsymbol{\beta}_0$	(4.700,-1.900,5.500,3.100)	(1.000,1.600,-2.700,1.100)	(1.700,2.200,-1.100,0.900)	(-3.800,2.300,1.700,2.100)
$\hat{\boldsymbol{\beta}}$	(4.699,-1.901,5.520,3.100)	(0.944,1.604,-2.697,1.101)	(1.687,2.215,-1.097,0.902)	(-3.804,2.304,1.699,2.099)
sd	[0.078,0.041,0.114,0.029]	[0.021,0.012,0.006,0.004]	[0.028,0.016,0.011,0.005]	[0.008,0.005,0.003,0.006]
$\boldsymbol{\beta}_0$	(0.700,2.900,-2.500,4.100)	(2.700,-1.200,2.600,0.900)	(3.500,3.700,6.700,-0.300)	(0.500,1.300,-0.700,2.300)
$\hat{\boldsymbol{\beta}}$	(0.688,2.902,-2.493,4,121)	(2.731,-1.233,2.604,0.901)	(3.394,3.722,6.880,-0.299)	(0.499,1.301,-0.700,2.301)
sd	[0.030,0.025,0.018,0.047]	[0.015,0.010,0.007,0.002]	[0.036,0.030,0.229,0.003]	[0.002,0.002,0.001,0.004]
$\boldsymbol{\beta}_0$	(2.200,-1.300,-1.400,2.200,1.600)	(2.100,-1.600,-1.200,2.900,-0.800)	(2.700,-1.300,1.600,2.100,-0.700)	(3.100,-1.000,-1.400,0.300,2.300)
$\hat{\boldsymbol{\beta}}$	(2.214,-1.297,-1.412,2.216,1.614)	(2.108,-1.604,-1.204,2.912,-0.804)	(2.725,-1.292,1.606,2.128,-0.701)	(3.097,-0.999,-1.399,0.302,2.300)
sd	[0.034,0.024,0.025,0.027,0.027]	[0.105,0.193,0.017,0.083,0.123]	[0.048,0.040,0.025,0.031,0.027]	[0.018,0.011,0.009,0.004,0.009]

[i] $\boldsymbol{\beta}_0$ is for the true value of $\boldsymbol{\beta}$

[ii] $\hat{\boldsymbol{\beta}}$ is for the estimates of $\boldsymbol{\beta}$

[iii] sd is for standard deviation of $\boldsymbol{\beta}$

Table 1. (Cont.)

	Shapes of $G(\cdot)$			
	Convex	*Concave*	$I_{t<0.5}$*Convex*+$I_{t\geq0.5}$*Concave*	$I_{t<0.2}$*Concave*+$I_{t\geq0.2}$*Convex*
$\boldsymbol{\beta}_0$	(-3.100,4.300,2.400,1.200,-1.600)	(-2.100,2.700,3.100,1.900,-0.600)	(1.900,1.300,-3.600,0.500,2.300)	(-1.100,2.300,1.700,0.900,-0.300)
$\hat{\boldsymbol{\beta}}$	(-3.101,4.348,2.385,1.210,-1.608)	(-2.102,2.708,3.103,1.905,-0.600)	(1.913,1.316,-3.623,0.507,2.318)	(-1.100,2.304,1.699,0.901,-0.300)
sd	[0.071,0.093,0.048,0.029,0.046]	[0.010,0.013,0.009,0.007,0.006]	[0.018,0.026,0.026,0.009,0.021]	[0.004,0.008,0.003,0.003,0.003]
$\boldsymbol{\beta}_0$	(3.600,-1.700,2.600,2.200,1.500)	(1.200,-1.500,0.300,2.400,-1.600)	(0.700,-2.100,1.000,1.500,1.300)	(-2.700,-0.700,-0.700,1.900,1.300)
$\hat{\boldsymbol{\beta}}$	(3.639,-1.703,2.609,2.230,1.530)	(1.202,-1.498,0.300,2.406,-1.603)	(0.709,-2.100,0.997,1.512,1.315)	(-2.701,-0.699,-0.702,1.903,1.303)
sd	[0.145,0.265,0.135,0.060,0.089]	[0.011,0.013,0.006,0.008,0.010]	[0.017,0.017,0.011,0.016,0.018]	[0.010,0.007,0.006,0.007,0.007]
$\boldsymbol{\beta}_0$	(-2.700,2.900,1.400,2.100,-2.300)	(-1.100,1.500,3.300,0.700,3.700)	(0.700,1.900,2.400,1.300,-1.900)	(1.100,-0.900,2.400,0.300,4.100)
$\hat{\boldsymbol{\beta}}$	(-2.706,2.924,1.399,2.110,-2.307)	(-1.151,1.497,3.356,0.701,3.743)	(0.701,1.944,2.400,1.318,-1.915)	(1.110,-0.900,2.384,0.300,4.128)
sd	[0.031,0.046,0.015,0.025,0.021]	[0.802,0.374,0.776,0.183,0.419]	[0.042,0.045,0.034,0.022,0.031]	[0.075,0.060,0.061,0.030,0.079]
	Case 2: $(I = 5000; J = 10)$			
$\boldsymbol{\beta}_0$	(2.300,3.500,1.800)	(1.200,2.900,0.600)	(2.400,2.300,1.600)	(1.700,2.100,1.200)
$\hat{\boldsymbol{\beta}}$	(2.301,3.503,1.798)	(1.200,2.899,0.600)	(2.403,2.306,1.598)	(1.701,2.103,1.199)
sd	[0.007,0.011,0.001]	[0.001,0.002,0.001]	[0.002,0.002,0.001]	[0.005,0.006,0.002]
$\boldsymbol{\beta}_0$	(-0.600,3.300,2.800)	(-1.100,2.900,1.800)	(-2.400,3.300,2.100)	(-0.900,2.300,3.400)
$\hat{\boldsymbol{\beta}}$	(-0.596,3.302,2.790)	(-1.099,2.901,1.799)	(-2.386,3.315,2.089)	(-0.899,2.302,3.393)
sd	[0.010,0.018,0.007]	[0.002,0.004,0.001]	[0.010,0.015,0.009]	[0.006,0.010,0.004]
$\boldsymbol{\beta}_0$	(-1.300,2.300,2.600)	(2.200,-2.300,3.600)	(2.500,-0.300,3.200)	(3.600,-0.400,4.100)
$\hat{\boldsymbol{\beta}}$	(-1.291,2.313,2.582)	(2.201,-2.225,3.533)	(2.503,-0.294,3.189)	(3.591,-0.403,4.089)
sd	[0.012,0.018,0.010]	[0.058,0.074,0.063]	[0.014,0.013,0.017]	[0.020,0.016,0.021]
$\boldsymbol{\beta}_0$	(1.400,5.300,-2.600)	(1.200,3.700,-1.800)	(2.600,1.500,-2.100)	(1.700,4.300,-2.700)
$\hat{\boldsymbol{\beta}}$	(1.402,5.303,-2.601)	(1.199,3.699,-1.800)	(2.601,1.508,-2.101)	(1.701,4.302,-2.700)
sd	[0.006,0.012,0.004]	[0.001,0.002,0.0005]	[0.006,0.007,0.003]	[0.003,0.005,0.002]
$\boldsymbol{\beta}_0$	(2.300,-0.600,2.400,3.100)	(-1.200,2.900,0.700,1.100)	(-0.300,1.200,2.100,2.100)	(2.800,-1.300,2.700,1.400)
$\hat{\boldsymbol{\beta}}$	(2.304,-0.605,2.398,3.092)	(-1.200,2.899,0.700,1.100)	(-0.306,1.205,2.100,2.100)	(2.828,-1.323,2.699,1.398)
sd	[0.005,0.003,0.003,0.019]	[0.001,0.001,0.0002,0.0005]	[0.002.0.001,0.001,0.002]	[0.009,0.006,0.010,0.008]
$\boldsymbol{\beta}_0$	(-3.900,3.600,-1.300,2.100)	(3.200,2.900,3.100,-1.100)	(3.300,-1.400,3.700,2.800)	(-4.100,3.300,2.600,2.400)
$\hat{\boldsymbol{\beta}}$	(-3.915,3.610,-1.299,2.090)	(3.181,2.910,3.095,-1.099)	(3.310,-1.409,3.701,2.802)	(-4.100,3.300,2.600,2.400)
sd	[0.013,0.015,0.001,0.015]	[0.011,0.008,0.006,0.001]	[0.004,0.003,0.001,0.002]	[0.001,0.001,0.001,0.002]
$\boldsymbol{\beta}_0$	(4.700,-1.900,5.500,3.100)	(1.000,1.600,-2.700,1.100)	(1.700,2.200,-1.100,0.900)	(-3.800,2.300,1.700,2.100)
$\hat{\boldsymbol{\beta}}$	(4.709,-1.912,5.490,3.093)	[0.997,1.602,-2.700,1.099)	(1.692,2.210,-1.100,0.901)	(-3.804,2.303,1.700,2.099)
sd	[0.009,0.005,0.035,0.017]	[0.002,0.001,0.0003,0.001]	[0.004,0.005,0.001,0.002]	[0.003,0.003,0.001,0.005]

Table 1. (Cont.)

	Shapes of $G(\cdot)$			
	Convex	*Concave*	$I_{t<0.5}$*Convex*+$I_{t\geq 0.5}$*Concave*	$I_{t<0.2}$*Concave*+$I_{t\geq 0.2}$*Convex*
$\boldsymbol{\beta}_0$	*(0.700,2.900,-2.500,4.100)*	*(2.700,-1.200,2.600,0.900)*	*(3.500,3.700,6.700,-0.300)*	*(0.500,1.300,-0.700,2.300)*
$\hat{\boldsymbol{\beta}}$	*(0.695,2.901,-2.498,4.100)*	*(2.730,-1.222,2.603,0.901)*	*(3.496,3.709,6.802,-0.300)*	*(0.499,1.300,-0.700,2.300)*
sd	*[0.003,0.003,0.001,0.020]*	*[0.009,0.006,0.004,0.002]*	*[0.006,0.008,0.121,0.0004]*	*[0.0005,0.0007,0.0001,0.004]*
$\boldsymbol{\beta}_0$	*(2.200,-1.300,-1.400,2.200,1.600)*	*(2.100,-1.600,-1.200,2.900,-0.800)*	*(2.700,-1.300,1.600,2.100,-0.700)*	*(3.100,-1.000,-1.400,0.300,2.300)*
$\hat{\boldsymbol{\beta}}$	*(2.206,-1.298,-1.405,2.206,1.606)*	*(2.104,-1.599,-1.203,2.905,-0.800)*	*(2.714,-1.294,1.602,2.115,-0.700)*	*(3.100,-1.000,-1.400,0.300,2.301)*
sd	*[0.017,0.011,0.015,0.018,0.015]*	*[0.008,0.006,0.0006,0.006,0.004]*	*[0.019,0.016,0.009,0.017,0.009]*	*[0.002,0.001,0.002,0.001,0.002]*
$\boldsymbol{\beta}_0$	*(-3.100,4.300,2.400,1.200,-1.600)*	*(-2.100,2.700,3.100,1.900,-0.600)*	*(1.900,1.300,-3.600,0.500,2.300)*	*(-1.100,2.300,1.700,0.900,-0.300)*
$\hat{\boldsymbol{\beta}}$	*(-3.099,4.316,2.394,1.203,-1.604)*	*(-2.102,2.706,3.102,1.903,-0.600)*	*(1.906,1.308,-3.610,0.503,2.309)*	*(-1.100,2.301,1.699,0.900,-0.300)*
sd	*[0.020,0.044,0.013,0.013,0.013]*	*[0.004,0.007,0.004,0.004,0.002]*	*[0.008,0.011,0.012,0.004,0.010]*	*[0.001,0.004,0.001,0.002,0.001]*
$\boldsymbol{\beta}_0$	*(3.600,-1.700,2.600,2.200,1.500)*	*(1.2,-1.5,0.3,2.4,-1.6)*	*(0.7,-2.1,1.0,1.5,1.3)*	*(-2.7,-0.7,-0.7,1.9,1.3)*
$\hat{\boldsymbol{\beta}}$	*(3.623,-1.696,2.603,2.218,1.519)*	*(1.201,-1.499,0.300,2.403,-1.601)*	*(0.703,-2.100,0.999,1.504,1.306)*	*(-2.700,0.699,-0.701,1.901,1.301)*
sd	*[0.059,0.046,0.037,0.035,0.041]*	*[0.004,0.004,0.002,0.004,0.002]*	*[0.004,0.005,0.003,0.006,0.007]*	*[0.003,0.002,0.002,0.004,0.003]*
$\boldsymbol{\beta}_0$	*(-2.700,2.900,1.400,2.100,-2.300)*	*(-1.100,1.500,3.300,0.700,3.700)*	*(0.700,1.900,2.400,1.300,-1.900)*	*(1.100,-0.900,2.400,0.300,4.100)*
$\hat{\boldsymbol{\beta}}$	*(-2.700,2.904,1.399,2.101,-2.301)*	*(-1.091,1.506,3.299,0.702,3.722)*	*(0.700,1.924,2.402,1.309,-1.910)*	*(1.105,-0.899,2.393,0.301,4.111)*
sd	*[0.006,0.015,0.002,0.007,0.006]*	*[0.107,0.128,0.067,0.041,0.119]*	*[0.012,0.022,0.008,0.009,0.009]*	*[0.018,0.025,0.022,0.006,0.029]*
	Case 3: $(I = 1000; J = 100)$			
$\boldsymbol{\beta}_0$	*(2.300,3.500,1.800)*	*(1.200,2.900,0.600)*	*(2.400,2.300,1.600)*	*(1.700,2.100,1.200)*
$\hat{\boldsymbol{\beta}}$	*(2.302,3.504,1.798)*	*(1.199,2.899,0.600)*	*(2.402,2.305,1.598)*	*(1.701,2.103,1.199)*
sd	*[0.005,0.009,0.001]*	*[0.001,0.001,0.0004]*	*[0.001,0.002,0.0005]*	*[0.003,0.004,0.001]*
$\boldsymbol{\beta}_0$	*(-0.600,3.300,2.800)*	*(-1.100,2.900,1.800)*	*(-2.400,3.300,2.100)*	*(-0.900,2.300,3.400)*
$\hat{\boldsymbol{\beta}}$	*(-0.595,3.306,2.792)*	*(-1.099,2.901,1.799)*	*(-2.390,3.312,2.091)*	*(-0.899,2.304,3.393)*
sd	*[0.007,0.013,0.005]*	*[0.003,0.004,0.002]*	*[0.008,0.014,0.007]*	*[0.004,0.006,0.003]*
$\boldsymbol{\beta}_0$	*(-1.300,2.300,2.600)*	*(2.200,-2.300,3.600)*	*(2.500,-0.300,3.200)*	*(3.600,-0.400,4.100)*
$\hat{\boldsymbol{\beta}}$	*(-1.294,2.311,2.582)*	*(2.168,-2.277,3.539)*	*(2.496,-0.303,3.188)*	*(3.592,-0.404,4.088)*
sd	*[0.011,0.019,0.007]*	*[0.025,0.044,0.046]*	*[0.010,0.009,0.012]*	*[0.014,0.011,0.015]*
$\boldsymbol{\beta}_0$	*(1.400,5.300,-2.600)*	*(1.200,3.700,-1.800)*	*(2.600,1.500,-2.100)*	*(1.700,4.300,-2.700)*
$\hat{\boldsymbol{\beta}}$	*(1.402,5.303,-2.601)*	*(1.199,3.698,-1.799)*	*(2.597,1.504,-2.100)*	*(1,701,4.302,-2.700)*
sd	*[0.004,0.008,0.003]*	*[0.0005,0.001,0.0002]*	*[0.004,0.004,0.002]*	*[0.002,0.003,0.001]*
$\boldsymbol{\beta}_0$	*(2.300,-0.600,2.400,3.100)*	*(-1.200,2.900,0.700,1.100)*	*(-0.300,1.200,2.100,2.100)*	*(2.800,-1.300,2.700,1.400)*
$\hat{\boldsymbol{\beta}}$	*(2.306,-0.605,2.399,3.099)*	*(-1.200,2.899,0.700,1.100)*	*(-0.306,1.205,2.100,2.099)*	*(2.828,-1.322,2.702,1.400)*
sd	*[0.002,0.001,0.001,0.003]*	*[0.0002,0.001,0.0002,0.0004]*	*[0.001,0.001,0.0004,0.001]*	*[0.003,0.002,0.002,0.002]*

Table 1. (Cont.)

	Shapes of $G(\cdot)$			
	Convex	*Concave*	$I_{t<0.5}$*Convex*+$I_{t\geq 0.5}$*Concave*	$I_{t<0.2}$*Concave*+$I_{t\geq 0.2}$*Convex*
$\boldsymbol{\beta}_0$	(-3.900,3.600,-1.300,2.100)	(3.200,2.900,3.100,-1.100)	(3.300,-1.400,3.700,2.800)	(-4.100,3.300,2.600,2.400)
$\hat{\boldsymbol{\beta}}$	(-3.915,3.612,-1.299,2.097)	(3.185,2.908,3.093,-1.100)	(3.310,-1.409,3.701,2.800)	(-4.100,3.300,2.600,2.400)
sd	[0.003,0.003,0.001,0.003]	[0.005,0.003,0.003,0.001]	[0.002,0.001,0.001,0.001]	[0.0005,0.0004,0.0003,0.001]
$\boldsymbol{\beta}_0$	(4.700,-1.900,5.500,3.100)	(1.000,1.600,-2.700,1.100)	(1.700,2.200,-1.100,0.900)	(-3.800,2.300,1.700,2.100)
$\hat{\boldsymbol{\beta}}$	(4.711,-1.911,5.502,3.099)	(0.997,1.602,-2.700,1.099)	(1.694,2.208,-1.100,0.901)	(-3.803,2.303,1.700,2.100)
sd	[0.004,0.002,0.009,0.004]	[0.001,0.001,0.0002,0.001]	[0.002,0.004,0.0004,0.002]	[0.001,0.001,0.0002,0.001]
$\boldsymbol{\beta}_0$	(0.700,2.900,-2.500,4.100)	(2.700,-1.200,2.600,0.900)	(3.500,3.700,6.700,-0.300)	(0.500,1.300,-0.700,2.300)
$\hat{\boldsymbol{\beta}}$	(0.697,2.901,-2.499,4.103)	(2.726,-1.220,2.601,0.900)	(3.498,3.705,6.766,-0.300)	(0.500,1.300,-0.700,2.300)
sd	[0.002,0.001,0.001,0.007]	[0.003,0.002,0.002,0.001]	[0.003,0.004,0.093,0.0003]	[0.0002,0.0002,0.0001,0.001]
$\boldsymbol{\beta}_0$	(2.200,-1.300,-1.400,2.200,1.600)	(2.100,-1.600,-1.200,2.900,-0.800)	(2.700,-1.300,1.600,2.100,-0.700)	(3.100,-1.000,-1.400,0.300,2.300)
$\hat{\boldsymbol{\beta}}$	(2.205,-1.300,-1.404,2.205,1.604)	(2.103,-1.600,1.202,2.904,-0.800)	(2.711,-1.298,1.603,2.112,-0.701)	(3.101,-1.000,-1.401,0.300,2.301)
sd	[0.012,0.004,0.009,0.012,0.010]	[0.005,0.005,0.004,0.006,0.003]	[0.016,0.008,0.006,0.012,0.004]	[0.002,0.001,0.001,0.001,0.002]
$\boldsymbol{\beta}_0$	(-3.100,4.300,2.400,1.200,-1.600)	(-2.100,2.700,3.100,1.900,-0.600)	(1.900,1.300,-3.600,0.500,2.300)	(-1.100,2.300,1.700,0.900,-0.300)
$\hat{\boldsymbol{\beta}}$	(-3.102,4.316,2.397,1.203,-1.604)	(-2.101,2.703,3.101,1.902,-0.600)	(1.904,1.304,-3.607,0.502,2.306)	(-1.100,2.301,1.700,0.900,-0.300)
sd	[0.015,0.035,0.009,0.010,0.008]	[0.004,0.005,0.003,0.003,0.002]	[0.006,0.005,0.008,0.003,0.008]	[0.001,0.002,0.001,0.001,0.0003]
$\boldsymbol{\beta}_0$	(3.600,-1.700,2.600,2.200,1.500)	(1.200,-1.500,0.300,2.400,-1.600)	(0.700,-2.100,1.000,1.500,1.300)	(-2.700,-0.700,-0.700,1.900,1.300)
$\hat{\boldsymbol{\beta}}$	(3.617,-1.702,2.605,2.214,1.513)	(1.200,-1.500,0.300,2.402,-1.601)	(0.703,-2.101,0.999,1.504,1.304)	(-2.700,-0.700,-0.700,1.901,1.301)
sd	[0.043,0.029,0.029,0.025,0.024]	[0.002,0.002,0.001,0.003,0.001]	[0.004,0.004,0.003,0.005,0.005]	[0.002,0.001,0.001,0.002,0.002]
$\boldsymbol{\beta}_0$	(-2.700,2.900,1.400,2.100,-2.300)	(-1.100,1.500,3.300,0.700,3.700)	(0.700,1.900,2.400,1.300,-1.900)	(1.100,-0.900,2.400,0.300,4.100)
$\hat{\boldsymbol{\beta}}$	(-2.701,2.905,1.400,2.102,-2.302)	(-1.096,1.507,3.298,0.702,3.713)	(0.701,1.915,2.402,1.306,-1.906)	(1.105,-0.901,2.396,0.300,4.111)
sd	[0.005,0.013,0.002,0.007,0.003]	[0.072,0.024,0.035,0.017,0.039]	[0.006,0.013,0.005,0.007,0.004]	[0.015,0.008,0.014,0.004,0.022]

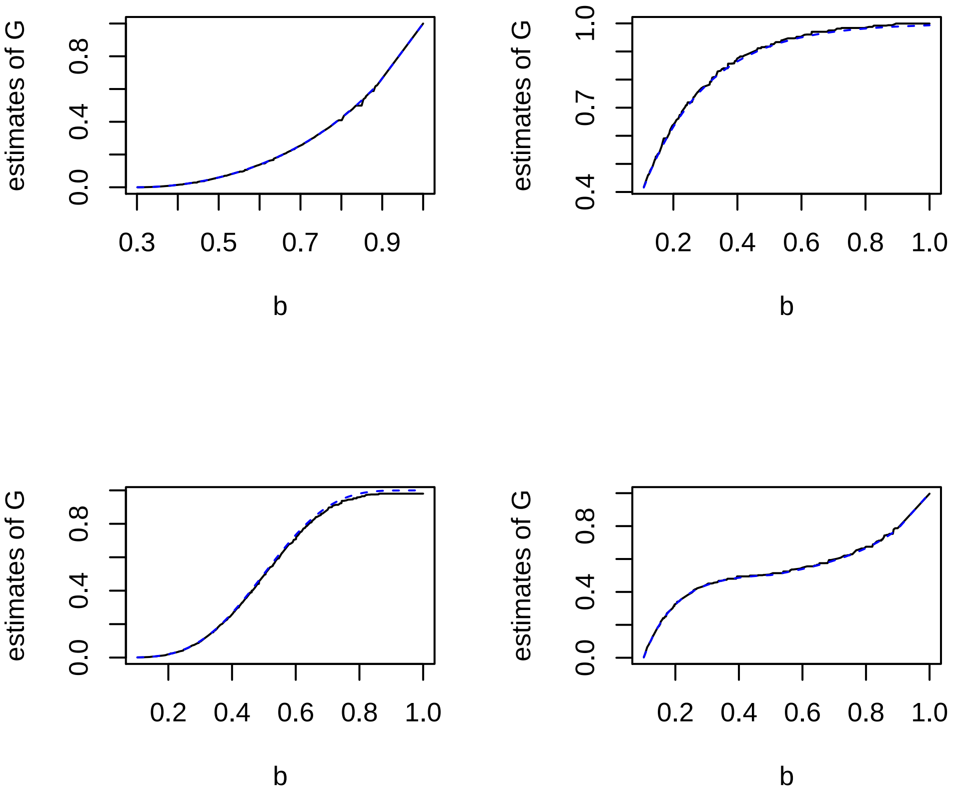

FIGURE 1 **Estimates of $G(t)$ when $G(t)$ is convex, concave, a mixture of convex and concave, sample size is** 200×10 **under Case 1: Solid line: True $G_0(\cdot)$; Step line: Estimate $\hat{G}(\cdot)$.**

| *Additional simulations: Robustness to different proportions of true zeros*

Above simulation settings in §2.6 are motivated by real examples in §2.7. Simulation studies in this subsection are to evaluate the robustness of the proposed method to different proportions of true zeros. We generate data with different proportions of true zeros.

TABLE 2 **Performance characteristic of the proposed method when the proportions of true zeros ($\rho(\%)$) and sample size varies.**

$\rho(\%)$	*Power(%)*	*Specificity(%)*	*Sensitivity(%)*	*FDR*
		I=200, J=10(True #=50,rr = 1.5)		

Table 2. (Cont.)

$\rho(\%)$	Power(%)	Specificity(%)	Sensitivity(%)	FDR
40	> 99.99	99.84	95.16	0.023
50	> 99.99	99.69	95.50	0.034
60	> 99.99	99.62	95.46	0.035
70	> 99.99	99.28	96.00	0.043
80	> 99.99	99.14	95.86	0.046
90	> 99.99	97.23	94.92	0.062
		I=1000, J=100(True #=350,$rr = 1.5$		
40	> 99.99	> 99.99	92.89	0.0002
50	> 99.99	> 99.99	93.06	0.0002
60	> 99.99	> 99.99	93.11	0.0001
70	> 99.99	> 99.99	93.50	0.0003
80	> 99.99	> 99.99	93.63	0.0004
90	> 99.99	> 99.99	94.27	0.005
		I=5000, J=10		
40	> 99.99	> 99.99	93.87	0.003
50	> 99.99	99.35	94.02	0.011
60	> 99.99	> 99.99	94.45	0.001
70	> 99.99	99.98	93.74	0.007
80	> 99.99	99.96	94.62	0.009
90	> 99.99	98.82	95.23	0.053

Table 2 shows that FDR increases as the proportion of true zeros increases. In general, the proposed method has high power, high specificity, and high sensitivity in all cases with different proportions of true zeros with controlling FDR in most cases (<0.05). When the sample size is small (200×10) and the proportion of true zeros is extremely large (90%), FDR is slightly larger than 0.05. The results demonstrate the robustness of the proposed method to different proportions of true zeros.

| *Additional simulations: Robustness to extreme weak signals*

Table 3 shows the results from the proposed method when signals are fragile ($rr < 1.5$). The proposed approach has high power and specificity when signals are extremely weak. However, the sensitivity is low when the signal strength is lower than 1.2. Still, it increases from 46.08% to 91.84% when the signal strength increases. One possible reason for low sensitivity, in this case, is that it is difficult to distinguish a weak signal from a noisy background.

TABLE 3 Performance characteristic of the proposed method when rr is extreme small and the signals are extreme weak.

True #	rr	Power(%)	Specificity(%)	Sensitivity(%)	FDR
		I=200, J=10			

TABLE 3 Performance characteristic of the proposed method when rr is extreme small and the signals are extreme weak.

True #	rr	*Power(%)*	*Specificity(%)*	*Sensitivity(%)*	*FDR*
50	*1.1*	> 99.99	*99.99*	*46.08*	*0.004*
50	*1.2*	> 99.99	*99.99*	*71.80*	*0.002*
50	*1.3*	> 99.99	*99.94*	*86.68*	*0.011*
50	*1.4*	> 99.99	*99.89*	*91.94*	*0.016*

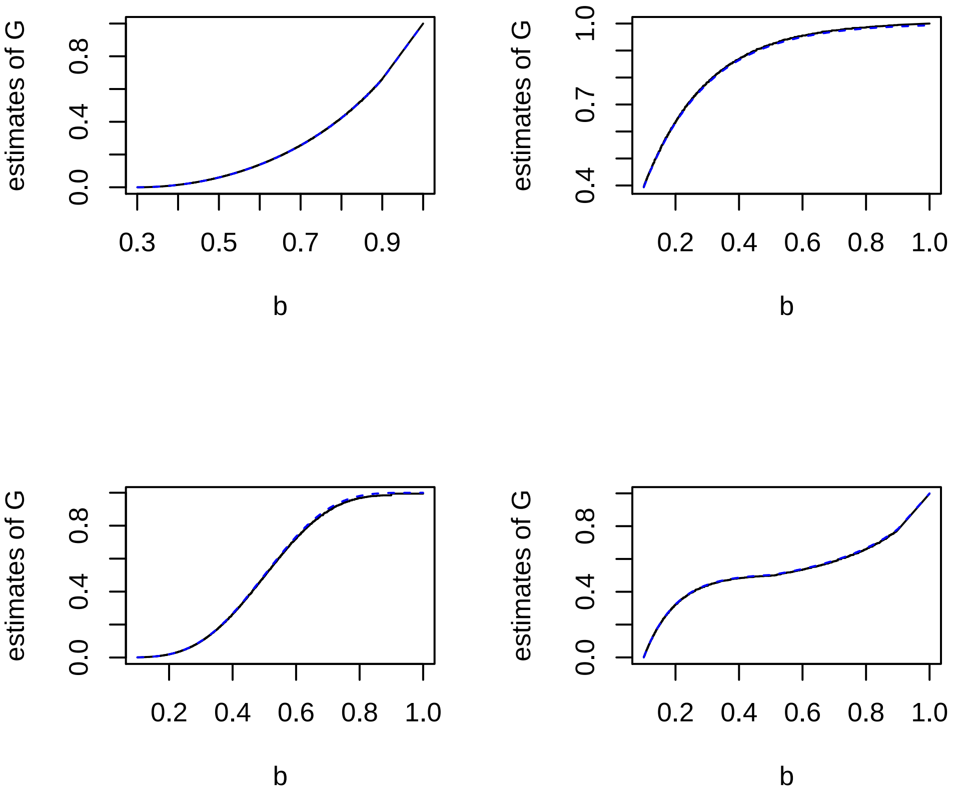

FIGURE 2 **Estimates of** $G(t)$ **when** $G(t)$ **is convex, concave, a mixture of convex and concave, sample size is** 5000×10 **under Case 2: Solid line: True** $G_0(\cdot)$**; Step line: Estimate** $\hat{G}(\cdot)$**.**

| Web Appendix C

| *Convergence and robustness across initialization scenarios*

To assess sensitivity, we conducted supplementary analyses using multiple, widely dispersed starting values for $\boldsymbol{\beta}^{(0)}$*. Across these runs, the algorithm consistently converged to the true value of* $\boldsymbol{\beta}$ *(within numerical tolerance), and the resulting parameter estimates and fit metrics remained effectively unchanged.*

We further investigated sensitivity through six simulation scenarios designed to probe the EM–isotonic procedure's dependence on initialization. These scenarios demonstrate that the choice of starting values does not affect the model's final

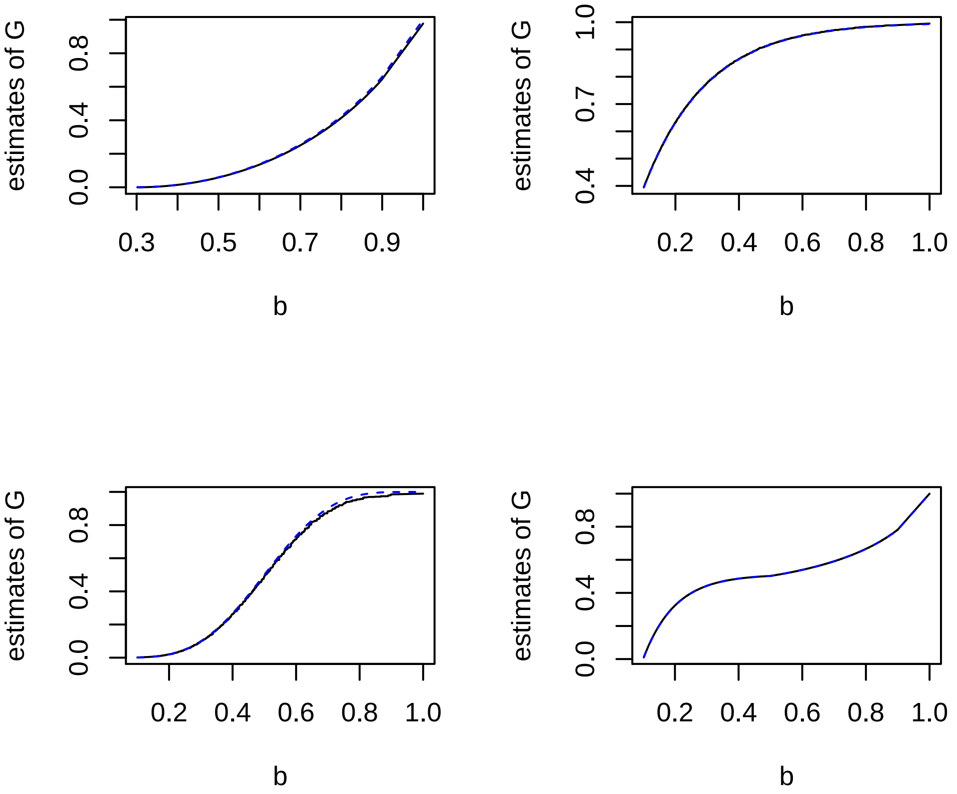

FIGURE 3 Estimates of $G(t)$ when $G(t)$ is convex, concave, a mixture of convex and concave, sample size is 1000×100 **under Case 3: Solid line: True $G_0(\cdot)$; Step line: Estimate $\hat{G}(\cdot)$.**

convergence (within numerical tolerance):

Scenario S1: $\boldsymbol{\beta}^{(0)}$ drawn randomly around $(0.84, 2.03, 0.42)$ *(approximately −30% relative to the true parameter value).*

Scenario S2: $\boldsymbol{\beta}^{(0)}$ drawn randomly around $(1.56, 3.77, 0.78)$ *(approximately +30% relative to the true parameter value).*

Scenario S3: $\boldsymbol{\beta}^{(0)}$ drawn randomly around $(0.48, 1.16, 0.24)$ *(approximately −60% relative to the true parameter value).*

Scenario S4: $\boldsymbol{\beta}^{(0)}$ drawn randomly around $(1.92, 4.64, 0.96)$ *(approximately +60% relative to the true parameter value).*

Scenario S5: $\boldsymbol{\beta}^{(0)}$ drawn randomly around $(1, 1, 1)$.

TABLE 4 **Convergence and robustness across initialization scenarios: $\boldsymbol{\beta}$ estimates, bias, and standard deviation**

Scenario	$\hat{\beta}$	Bias	SD
S1	(1.20,2.90,0.60)	<0.001	(0.002,0.003,0.003)
S2	(1.20,2.90,0.60)	<0.001	(0.002,0.003,0.003)
S3	(1.20,2.90,0.60)	<0.001	(0.002,0.003,0.003)
S4	(1.20,2.90,0.60)	<0.001	(0.002,0.003,0.003)
S5	(1.20,2.90,0.60)	<0.001	(0.002,0.003,0.003)
S6	(1.21,2.92,0.59)	(0.01,0.02,-0.01)	(0.007,0.010,0.005)

Notes: Results are based on 1000 simulation replicates with $I = 200$ and $J = 10$ under a concave true $G(\cdot)$ and true parameter value $\boldsymbol{\beta} = (1.2, 2.9, 0.6)$.

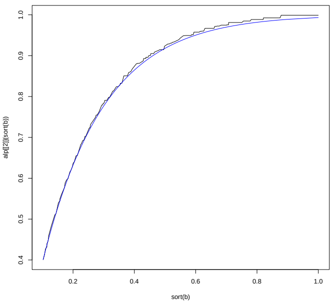

Scenario S1

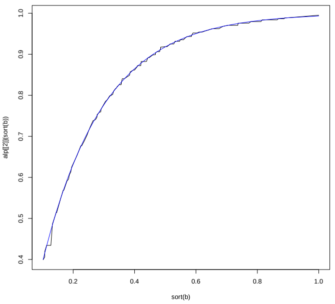

Scenario S2

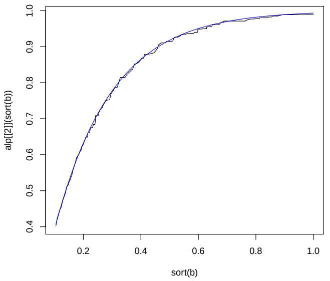

Scenario S3

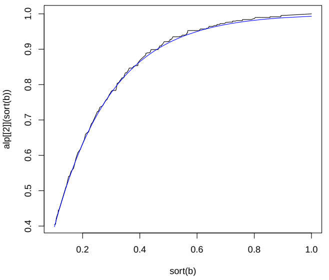

Scenario S4

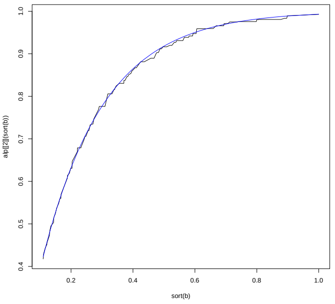

Scenario S5

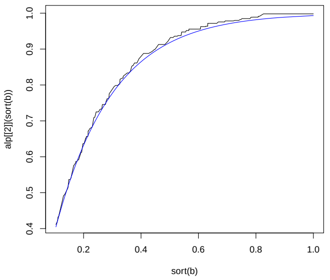

Scenario S6

FIGURE 4 **Estimated $G(\cdot)$ across six initialization scenarios: Solid line: True $G_0(\cdot)$; Step line: Estimate $\hat{G}(\cdot)$.**

Scenario S6: $\boldsymbol{\beta}^{(0)}$ drawn randomly around $(-1, -1, -1)$.

For illustration, in the case with $I = 200$, $J = 10$, a concave true $G(\cdot)$, and true parameter value $\boldsymbol{\beta} = (1.2, 2.9, 0.6)$, the algorithm converged to the same solution across independent runs; each scenario was repeated 1,000 times. We report the simulation results for all scenarios in Table 4 and present the corresponding estimates of $G(\cdot)$ in Figure 4.

From Table 4 and Figure 4, the estimates of $(\boldsymbol{\beta}, G(\cdot))$ *remain stable as* $(\boldsymbol{\beta}^{(0)}, G^{(0)})$ *are varied. The bias and SD show no evident trend across initialization scenarios.*

references

[1] van der Vaart A, Wellner J. Weak Convergence and Empirical Processes. Springer; 1996.

[2] Bickel PJ, Klaassen CA, Ritov Y, Wellner JA. Efficient and Adaptive Estimation for Semiparametric Models. Baltimore: Johns Hopkins University Press; 1993.

[3] van der Vaart A. Semiparametric Statistics. In: Lecture Notes in Mathematics, Ed. Cachan, J.M.M., Groningen, F.T., Paris, B.T. Springer; 2002.
